# Peculiar Differences between Two Copper Complexes Containing Similar Redox-Active Ligands: Density Functional and Multiconfigurational Calculations

**DOI:** 10.1021/acs.inorgchem.3c02949

**Published:** 2023-12-29

**Authors:** Luca Gerhards, Marco Werr, Olaf Hübner, Ilia A. Solov’yov, Hans-Jörg Himmel

**Affiliations:** † Institute of Physics, Carl von Ossietzky Universität Oldenburg, Carl-von-Ossietzky-Street 9-11, Oldenburg 26129, Germany; ‡ Anorganisch-Chemisches Institut, Ruprecht-Karls-Universität Heidelberg, Im Neuenheimer Feld 270, Heidelberg 69120, Germany; § Research Center for Neurosensory Science, Carl von Ossietzky Universität Oldenburg, Oldenburg 26111, Germany; ∥ Center for Nanoscale Dynamics (CENAD), Carl von OssietzkyUniversität Oldenburg, Institut Für Physik, Ammerländer Heerstreet 114-118, Oldenburg 26129, Germany

## Abstract

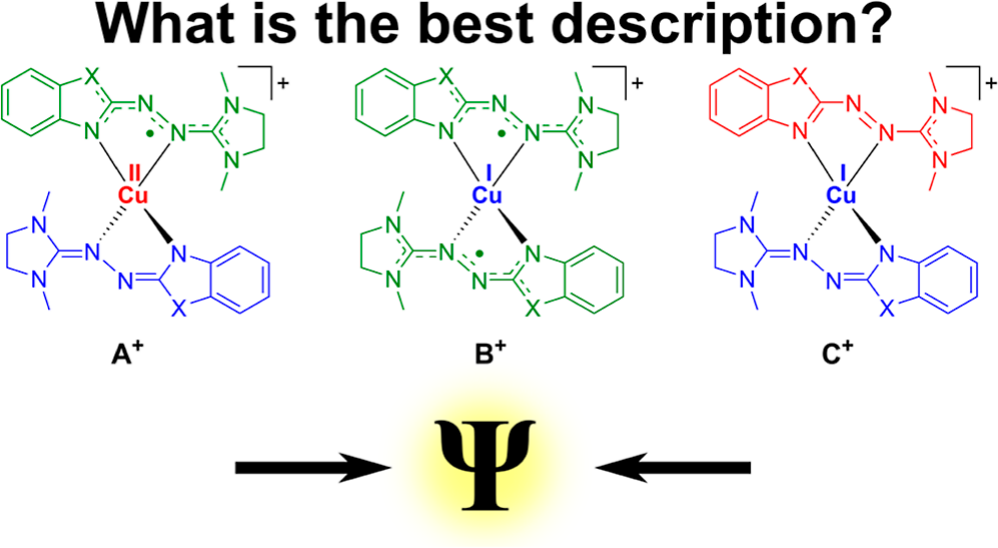

Transition metal complexes featuring redox-active ligands often exhibit multiple redox states, influenced by the interplay between the metal center and the ligand. This study delves into the electronic structures of two mononuclear complexes of copper with two similar redox-active urea azine ligands. The ligands differ by the replacement of an NCH_3_ moiety by an S atom in the ligand backbone. Experimental analysis yields pronounced electronic structural disparities between these complexes, observable in both the solution and solid phases. Conventional quantum chemical methods, such as density functional theory using different functionals (B3LYP, TPSSh, and CAM-B3LYP), remain inadequate to rationalize the observed spectroscopic anomalies. However, a multiconfigurational approach elucidates the disparate behaviors of these complexes. Multireference perturbation theory, based on complete active space self-consistent field computations, identifies Cu(I) in the case of the complex with the NCH_3_ containing ligands and a state with substantial Cu(II) contributions in the case of the complex with the S atom containing ligands. In contrast, DFT indicates Cu(I) in both scenarios.

## Abstract 2

The investigation of the electronic structure of oxidized mononuclear homoleptic tetrahedral copper complexes reveals an underlying complexity which can only be described by multiconfigurational quantum chemistry methods. Moreover, several electronic structures are involved and can be favored through the use of different functional groups.

## 1 Introduction

Redox-active ligands are easily oxidized or reduced compared to classical spectator ligands.^1−13^ Such ligands are a means to extend the redox reactivity of metal atoms in complexes because they are able to provide additional redox equivalents. Usually, the number of electrons that a mononuclear metal complex can transfer to a substrate is limited to one or two, but in complexes with redox-active ligands, the ligands may deliver additional electrons.

A special property of complexes composed of redox-active metals and redox-active ligands is the ability of intramolecular electron transfer (IET), allowing the formation of bistable systems^14^ exhibiting electromeric structures. Prime examples are octahedral cobalt complexes with two redox-active dioxolene-type ligands.^15^ In these complexes, an intramolecular metal–ligand electron transfer (combined with spin crossover) could be stimulated by temperature or by light.^14^ The design of complexes that are stable in two or more electromeric forms and that can be interconverted reversibly by an external stimulus is of considerable interest for applications, e.g., in molecular-level switching and information storage devices.^14^ Therefore, a profound understanding of the electronic structure and how it affects reactivity and physical properties is desirable for the rational design of such complexes.

Interestingly, an IET can also be triggered by a redox event. The overall one-electron oxidation of a molecular complex may lead to a one-electron reduction of a part of it or vice versa. This so-called redox-induced electron transfer (RIET) was first discovered by Miller et al. in 2007 for dinuclear cobalt tetraoxolene complexes.^16^ Further systems were discussed in a review article in 2009.^17^ Also, the relevance of RIET in biological systems was highlighted, e.g., the reduction of Cytochrome *b* upon aerobic oxidation,^18,19^ the functioning of Ni–Fe hydrogenase,^17^ and also the limitations of a theoretical treatment for describing RIET were discussed.

**Figure 1:**
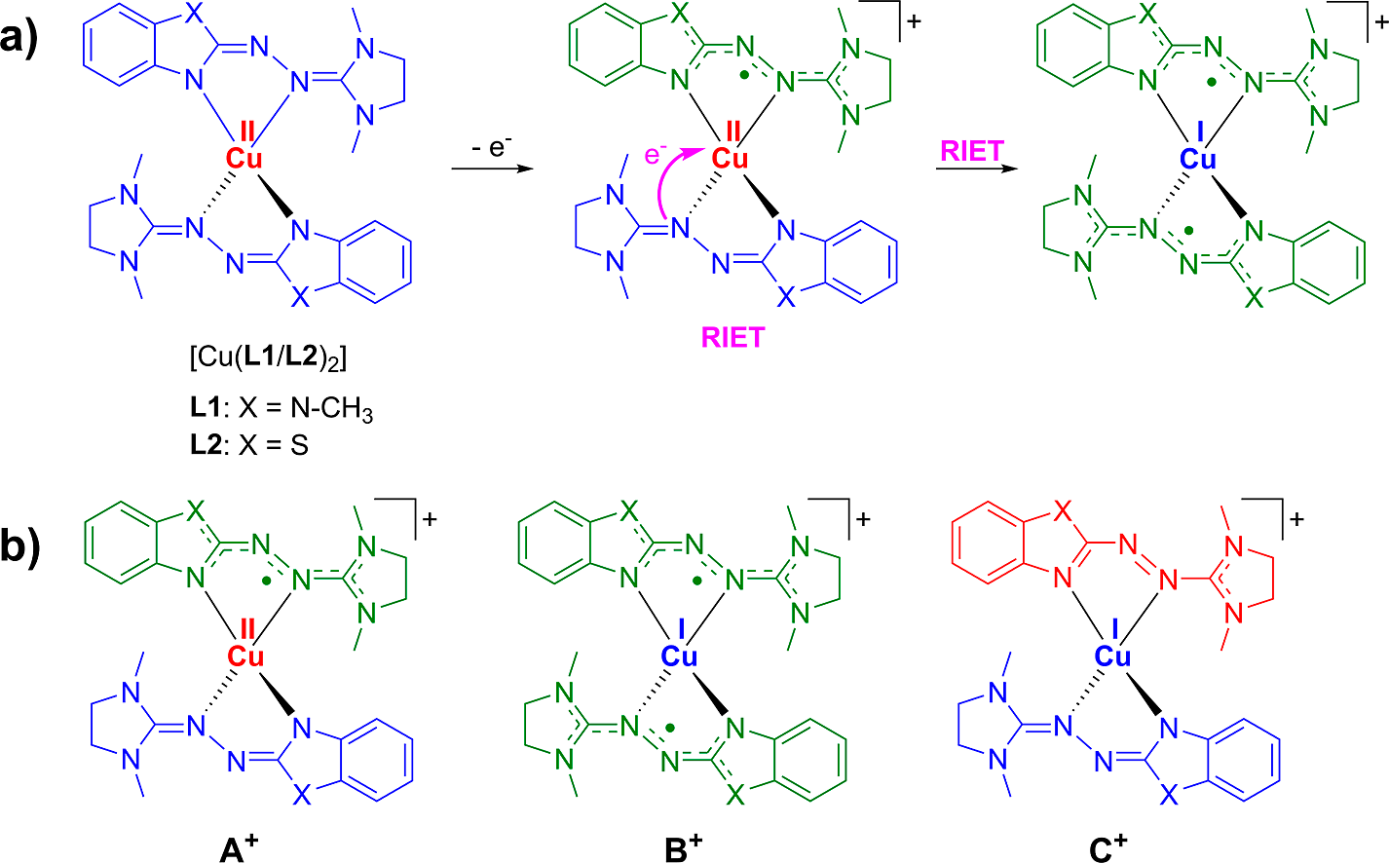
(a) Lewis structures of the two neutral Cu complexes and a schematic representation of the RIET process upon their one-electron oxidation. After an electron is extracted from the molecule (blue to green), electron density from the ligands is transferred to the metal center (metal: red to blue, ligand: blue to green). The amount of electron density donated to the metal center depends on the functional groups of the ligands. (b) Proposed electronic structures of the monocationic complexes [Cu(L1)_2_]^+^ and [Cu(L2)_2_]^+^. The unpaired ligand-centered electron in structure **A**^**+**^ could be either located on one ligand **A**_asym_^+^ or delocalized over both ligands **A**_sym_^+^.

**Figure 2:**
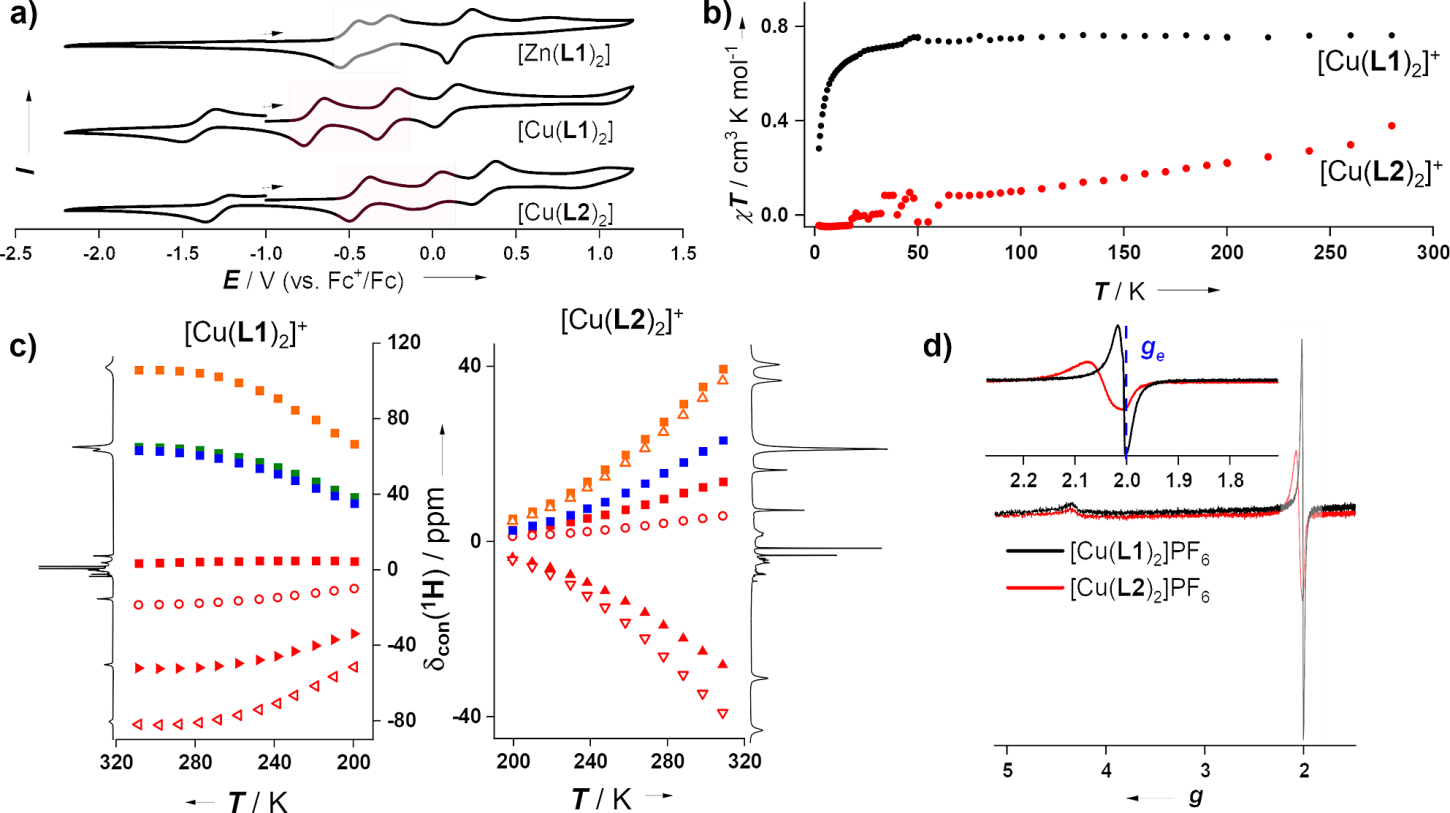
Comprehensive experimental overview of the homoleptic urea azine complexes of copper (Cu) and zinc (Zn) with the ligands L1 and L2. This figure is divided into four main sections: (a) cyclic voltammograms: The anodic scan is performed at 100 mV s^–1^ in CH_2_Cl_2_, and the results are referenced externally to the redox couple ferrocenium/ferrocene (Fc^+^/Fc). The redox waves, which signal the stabilization of the oxidized, monocationic form of the copper complexes, are highlighted in pink. (b) Plot of the magnetometric data, χ*T*, versus temperature, obtained using a SQUID.^32^ The data for the complexes [Cu(L1)_2_]SbF_6_ and [Cu(L2)_2_]PF_6_ are measured at 50 mT, revealing a significant paramagnetic behavior for [Cu(L1)_2_]^+^ and a less paramagnetic nature for [Cu(L1)_2_]^+^ at higher temperatures. (c) Evolution of the extracted ^1^H NMR contact shifts with temperature (399.89 MHz, CD_2_Cl_2_) for the complexes [Cu(L1/L2)_2_]PF_6_. A notable deviation of the paramagnetic shift with respect to the temperature is observed for both complexes. (d) Solid-state X-band EPR spectra of [Cu(L1)_2_]PF_6_ and [Cu(L2)_2_]PF_6_ at 6.2 and 6.3 K, respectively. It includes a full scan and a magnified view near the free-electron *g* factor (*g*_e_ = 2.0023). A stronger deviation from the free-electron *g* factor is evident in [Cu(L2)_2_]PF_6_. For additional details on the experimental data, refer to the cited literature.^27^

Another example of a class of complexes that can exhibit electromerism are the complexes of first-row transition metals with bis- and tetrakis-guanidino-functionalized aromatic ligands. Studies of complexes with these redox-active ligands revealed a low barrier for ligand–metal electron transfer processes.^20−23^ In continuation of that work, new redox-active urea azine ligands^24−27^ were developed, and their copper complexes proved to be catalysts for the selective aerobic oxidation of organic substrates,^27^ in which the redox-active ligands contribute to the required number of electrons. Moreover, for some of these complexes, a RIET process was observed upon oxidation.^27^ For the similar neutral mononuclear homoleptic tetrahedral copper complexes [Cu(L1)_2_] and [Cu(L2)_2_] sketched in Figure 1a, with the two different urea azine ligands L1 and L2 (L1 being an urea azine ligand with an NCH_3_ group, L2 being a thio-urea azine ligand with an S atom in place of the NCH_3_ group), the one-electron oxidation induces a RIET. However, the electronic structure of the resulting cationic complexes [Cu(L1)_2_]^+^ and [Cu(L2)_2_]^+^ still remains unclear. Especially, several experiments revealed significant differences between the two complexes (see Figure 2).

The Lewis structures sketched in Figure 1b illustrate possible postulated electronic states of [Cu(L1)_2_]^+^ and [Cu(L2)_2_]^+^. Note that the Lewis structures are simplified representations of the true electronic states but are useful to depict the essential characteristics of the respective states. Structure **A**^**+**^ is a complex of Cu(II) with one neutral radical ligand and a second monoanionic ligand. However, the unpaired electron could also be delocalized over both ligands. Structures **B**^**+**^ and **C**^**+**^ are complexes of Cu(I). In **B**^**+**^, both ligands are neutral radicals. In the case of **C**^**+**^, the two ligands are in two different closed-shell states, one in a monoanionic state and the other in a monocationic one. In our previous work,^27^ it was shown that the experimental observations could not be explained by only one of these structures, and it was postulated that both structures **B**^**+**^ and **C**^**+**^ contribute to the resulting electronic state of the monocationic complexes but that the weight of the two structures would be very different for [Cu(L1)_2_]^+^ and [Cu(L2)_2_]^+^.

In this context, it is worth mentioning that ruthenium complexes of redox-active azo-derived 2,2′-azobis(benzothiazole) (abbt) and azobis(1-methylbenzimidazole) (abim) ligands, structurally related to the present ones, have been studied by Panda, Lahiri et al.^28^ These types of ligands can be understood as twice-deprotonated, symmetric (thio)urea azines and represent an inverted redox system compared to the presently studied urea azines.^29−31^ These ligands show a similar coordination mode, but the shifted charge range (−2, −1, 0 vs −1, 0, +1 for the urea azines) after double-deprotonation often leads to the formation of binuclear complexes.

In this work, the goal is to understand the electronic structures of the two cationic copper complexes [Cu(L1)_2_]^+^ and [Cu(L2)_2_]^+^ and to explain the observed differences in the experiments. The rational approach followed in this work should be applicable to other systems and therefore contribute toward a better understanding of the electronic structures of complexes with redox-active ligands in general. At first, a brief summary of the previous experimental findings is given as the basis for the further theoretical investigations. Furthermore, the computational results using density functional theory (DFT) with different functionals, e.g., B3LYP and TPSSh, as well as multiconfigurational wave function methods, are presented. Based on this combined investigation, it will be possible to explain the ambivalent behavior of the two urea azine ligands and the observed variation of the electronic structure with temperature. Additionally, it is possible to make predictions for further directed manipulations of the systems.

## 2 Results

### 2.1 Former and New Experiments

Before we turn to the results of the novel quantum-chemical calculations, a summary of the experimental results for the two monocationic complexes [Cu(L1)_2_]^+^ and [Cu(L2)_2_]^+^ is given to facilitate the discussion. The synthesis and most of the experimental data were already presented in an earlier study,^27^ but we added some new measurements (IR spectra and new electron paramagnetic resonance (EPR) spectra as well as new superconducting quantum interference device (SQUID) data). These results clearly show that the two complexes show unexpected differences in their electronic structures.

The two neutral complexes [Cu(L1)_2_] and [Cu(L2)_2_] were prepared from Cu(II) acetate and the neutral, protonated urea azines HL1 and HL2, respectively.^27^ The redox behavior of the two complexes was studied by cyclic voltammetry (CV) and compared to that of the corresponding zinc urea azine complex [Zn(L1)_2_] with the redox-inactive zinc (see Figure 2a). In the CV experiments, the anodic scans show three reversible one-electron redox events for the analogue complexes [Zn(L1)_2_] and [Cu(L1)_2_]. However, the potential difference between the first and second redox events is larger in the case of the copper complexes. Hence, a higher stability toward disproportionation of the monocationic state into the neutral and dicationic states can be inferred for the copper complex. Further experiments showed that the stabilization can be rationalized by a redox-induced IET (RIET) upon oxidation (see Figure 1a).^27^ This likely is caused by the redox-active copper atom interacting with the redox-active urea azine ligands. Comparing the redox behavior of [Cu(L1)_2_] and [Cu(L2)_2_], a similar potential difference between the first and second redox events is observed. However, the redox waves in the CV of [Cu(L2)_2_] are shifted toward a higher potential compared to [Cu(L1)_2_]. This shift is in line with the higher redox potential of the uncoordinated, protonated ligand HL2 compared to HL1.

Subsequently, the monocationic complexes [Cu(L1)_2_]^+^ and [Cu(L2)_2_]^+^ were generated by chemically oxidizing the neutral complexes with ferrocenium hexafluorophosphate [Fc(PF_6_)]. Intriguingly, the characterization of the two oxidized, monocationic complexes [Cu(L1)_2_]^+^ and [Cu(L2)_2_]^+^ revealed significant differences in their solid-state structures and spectroscopic properties. For example, the investigation of the magnetic properties of [Cu(L1)_2_]^+^ in the solid state via SQUID magnetometry (see Figure 2b) shows a behavior of χ*T* as a function of the temperature that is typical for a weakly antiferromagnetically coupled diradical.^27^ At 280 K, χ*T* approaches the spin-only value for two electrons (0.75 cm^3^ K mol^–1^). By contrast, in the case of [Cu(L2)_2_]^+^, χ*T* increases only slowly with the temperature, approaching a value of just 0.36 cm^3^ K mol^–1^ at 280 K.^32^ For both complexes, the magnetometric data argue for a singlet ground state, but the considerably slower increase of χ*T* for [Cu(L2)_2_]^+^ points to a larger energy difference between the diamagnetic singlet and a paramagnetic triplet state.

**Figure 3:**
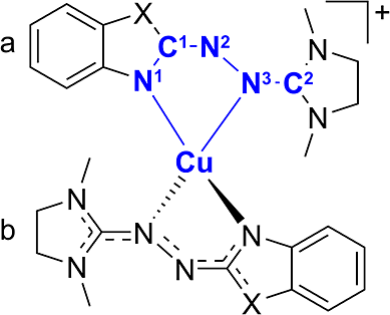
Nomenclature of atoms (blue) involved in the bonds considered in Table 1. The X (=NCH_3_, S) illustrates the functional group that differs between the [Cu(L1)_2_] and [Cu(L2)_2_] complexes.

Also, the crystal structures of the two copper complexes in their neutral and oxidized states reveal differences in the structures of the ligands (see Table 1 and Figure 3). There are two identical L1 ligands in the solid state structure of [Cu(L1)_2_]SbF_6_ with harmonization of the azine C^1^–N^2^–N^3^–C^2^ bond lengths compared to the neutral [Cu(L1)_2_] complex. In addition, one of the Cu–N^1,3^ bond lengths increases significantly upon oxidation, leading to a larger average Cu–N bond length compared to the neutral complex. The dihedral angle between the N–Cu–N planes of each ligand is also increased (from 51.5 to 65.4°),^27^ arguing for a higher occupation of the Cu 3d orbitals and supporting a description rather in terms of a Cu(I) atom and two oxidized, neutral radical ligands. On the other hand, in the solid-state structure of [Cu(L2)_2_]SbF_6_, the copper atom is coordinated by two clearly different L2 ligands. Similar to the L1 complex, for both of the two L2 ligands, the bond lengths within the central C^1^–N^2^–N^3^–C^2^ units of the ligands harmonize (see Table 1), but the change is clearly smaller for one of the ligands. Furthermore, the two Cu–N^1,3^ bonds in [Cu(L2)_2_]^+^ are shortened on average compared to the neutral [Cu(L2)_2_] complex, in contrast to the [Cu(L1)_2_]^+^ complex, where one Cu–N^1,3^ distance is strongly elongated. The dihedral angle between the N–Cu–N planes of each ligand is increased through oxidation but slightly less than for [Cu(L1)_2_]SbF_6_ (from 54.0 to 60.9°).^27^ Overall, the structural data point to a higher oxidation state of the copper atom in [Cu(L2)_2_]SbF_6_ than in [Cu(L1)_2_]SbF_6_.

**Table 1:** Comparison between Selected X-ray diffraction Bond Lengths [Å] of [Cu(L1/L2)_2_]SbF_6_ and Their Neutral, Reduced Forms [Cu(L1/L2)_2_]^a^

complex	bond	ligand a	ligand b
[Cu(L1)_2_]SbF_6_	Cu–N^1^	1.993(3)	1.993(3)
	Cu–N^3^	2.095(3)	2.095(3)
	C^1^–N^2^	1.340(5)	1.340(5)
	N^2^–N^3^	1.369(4)	1.369(4)
	N^3^–C^2^	1.341(5)	1.341(5)
[Cu(L1)_2_]	Cu–N^1^	1.924(2)	1.924(2)
	Cu–N^3^	2.007(2)	1.976(2)
	C^1^–N^2^	1.305(2)	1.307(2)
	N^2^–N^3^	1.426(2)	1.426(2)
	N^3^–C^2^	1.313(2)	1.313(2)
[Cu(L2)_2_]SbF_6_	Cu–N^1^	1.909(3)	1.922(3)
	Cu–N^3^	1.962(3)	1.986(3)
	C^1^–N^2^	1.310(4)	1.327(4)
	N^2^–N^3^	1.405(4)	1.371(4)
	N^3^–C^2^	1.340(4)	1.362(4)
[Cu(L2)_2_]	Cu–N^1^	1.944(2)	1.944(2)
	Cu–N^3^	1.985(2)	1.985(2)
	C^1^–N^2^	1.304(3)	1.304(3)
	N^2^–N^3^	1.420(2)	1.420(2)
	N^3^–C^2^	1.326(3)	1.326(3)

a Bonds are visualized in Figure 3. Ligands a and b correspond to the two different coordinated ligands within each of the complexes.

A comparison of newly measured ATR-IR powder spectra of the [Cu(L1/L2)_2_]PF_6_ complexes with the spectra obtained for the neutral reduced forms (see Supporting Information, Figure S2) reveals a clear shift of the asymmetric stretching modes  (C=N) of [Cu(L1)_2_]PF_6_ (ca. 1530 cm^–1^) compared to neutral [Cu(L1)_2_] (ca. 1568 cm^–1^), whereas these modes are almost identical for [Cu(L2)_2_]PF_6_ (ca. 1531 cm^–1^) and [Cu(L2)_2_] (ca. 1529 cm^–1^).

The evaluation of the temperature-dependent magnetic behavior in solution via ^1^H nuclear magnetic resonance (NMR) spectroscopy (see Figure 2c) shows a non-Curie behavior of the extracted contact shifts (decrease of the contact shift with decreasing temperature) for both monocationic complexes [Cu(L1/L2)_2_]^+^, but the effect is much stronger for [Cu(L2)_2_]^+^. The results argue for an antiferromagnetic interaction (singlet ground state) in both complexes but a stronger magnetic coupling in [Cu(L2)_2_]^+^.^27^ Most importantly, the magnitude of the paramagnetic shifts of the signals is significantly larger for [Cu(L1)_2_]^+^, indicating a higher spin density on the ligands.

New EPR measurements of solid state samples at the low-temperature limit (see Figure 2d and Supporting Information, Figure S1; for previous EPR spectra in solution, see ref (27)) found a weak transition (Δ*m*_s_ = 2) in the half-field region (*g* ≃ 4.3) for both complexes [Cu(L1/L2)_2_]PF_6_. The observation of a half-field signal clearly shows the presence of two unpaired electrons in the monocationic complexes. In addition, for both complexes, a signal close to the value of the free electron (*g*_e_ = 2.0023) is observed. The value is higher for [Cu(L2)_2_]PF_6_ (*g* = 2.0451) than for [Cu(L1)_2_]PF_6_ (*g* = 2.0050), arguing for an unpaired electron partly localized at the copper atom of the former complex, as indicated by an increase of the isotropic *g* value, presumably through spin–orbit coupling. The comparison of the *g* values of solid state EPR spectra with the values for frozen solutions^27^ ([Cu(L1)_2_]PF_6_: *g* = 2.0069, [Cu(L2)_2_]PF_6_: *g* = 2.0350) reveals qualitative agreement: For the L1 complex, the values are close to the free electron *g* value; for the L2 complex, there is a clear deviation from the value of the free electron, but the difference is somewhat more pronounced in the solid state (see Supporting Information, Figure S1.)

The UV–vis spectra of the neutral complexes [Cu(L1)_2_] and [Cu(L2)_2_] display weak absorption bands in the visible region and a very weak band in the near-infrared (NIR), around 1500 nm.^27^ Upon one-electron oxidation, the green complexes turn purple. The UV–vis spectra of the monocationic complexes contain strong absorption bands in the visible region and significantly stronger absorptions in the NIR region (see Figure 5). The spectra argue for the presence of ligand-centered radicals and facile intramolecular charge-transfer processes due to mixed valency. In the earlier study,^27^ a comparison of the observed absorption spectra for [Cu(L1)_2_]^+^ with spectra obtained by TDDFT calculations on the free L1 radical ligand suggested the presence of two unpaired electrons on the ligands.

**Figure 4:**
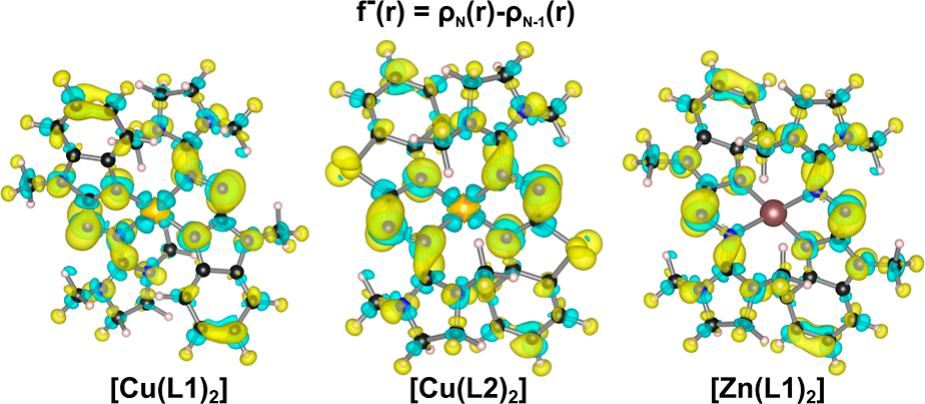
Fukui function plot *f*^–^(**r**) for [Cu(L1)_2_], [Cu(L2)_2_], and [Zn(L2)_2_] (B3LYP/def2-TZVP).^41,42^ A negative value (cyan color) means a gain of electron density, and a positive value (yellow color) means a loss of electron density upon oxidation.

**Figure 5:**
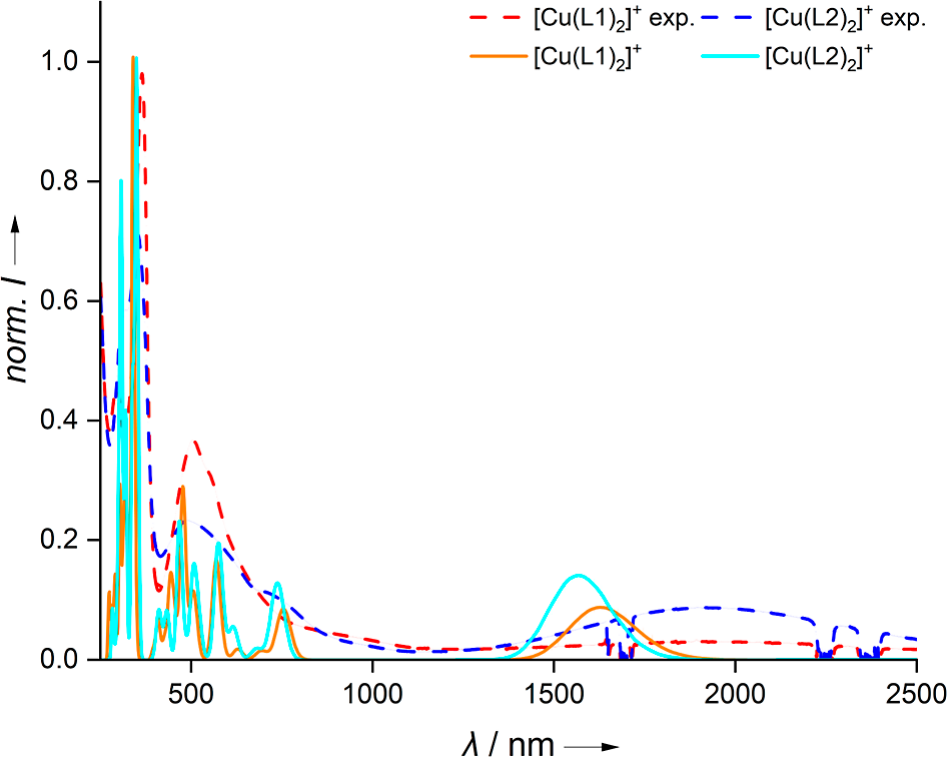
Electronic excitation spectra by TDDFT calculations (B3LYP/def2-TZVP) for the triplet states of the [Cu(L1)_2_]^+^ and [Cu(L2)_2_]^+^ complexes at the structures obtained with inclusion of the D3 dispersion correction (B3LYP + D3/def2-TZVP) and experimental UV–vis spectra of the salts [Cu(L1)_2_]PF_6_ and [Cu(L2)_2_]PF_6_, measured in CH_2_Cl_2_.^27^ The TDDFT calculations determined the 100 lowest roots. The calculated transitions were fitted with Gaussians of 0.1 eV width.

To conclude, the experimental data shows that both complexes have open-shell singlet ground states, but nevertheless, the electronic situations of the two complexes clearly differ. The [Cu(L1)_2_]^+^ complex appears to be better described as a Cu(I) complex with two weakly antiferromagnetically coupled urea azine radical ligands (corresponding to structure **B**^**+**^ of Figure 1b) with vanishing spin density on the Cu atom. On the other hand, the [Cu(L2)_2_]^+^ complex shows a relatively strong antiferromagnetic coupling and some spin density on the Cu atom, arguing for at least a partial Cu(II) character (in accordance with contributions of structure **A**^**+**^). Furthermore, the two different ligands in the crystal structure of [Cu(L2)_2_]^+^ point to structure **A**_asym_^+^ (see Figure 1b). Hence, quantum-chemical calculations seem to be advised to explain the differences.

### 2.2 Density Functional Calculations

Density functional calculations within the broken-symmetry approach are able to approximately treat open-shell singlet electronic states in many cases.^33−36^ Therefore, the electronic structures of both the [Cu(L1)_2_]^+^ and [Cu(L2)_2_]^+^ complexes were probed first via density functional calculations. However, the accurate treatment of different spin states by density functional methods in general can be problematic and, in particular, depend on the amount of Hartree–Fock exchange incorporated into the functional.^37^ Therefore, among others (see Supporting Information, Table S1), two functionals that differ in the amount of Hartree–Fock exchange have been employed. Initially, for both complexes, structure optimizations have been performed both with and without inclusion of the D3 dispersion correction (see Supporting Information, Table S7). The structures without D3 correction show significantly larger deviations from the crystal structures and are not further considered. In general, the density functional optimizations reveal symmetric structures for both complexes.

The B3LYP density functional calculations (including the D3 dispersion correction) yield triplet ground states (^3^A) for both complexes, and the broken-symmetry states (^BS^A) are only slightly higher in energy at 0.012 ([Cu(L1)_2_]^+^) and 0.023 ([Cu(L2)_2_]^+^) eV (Table 2). This is also reflected in the J-couplings, as can be observed in Table 2. By contrast, the closed-shell singlet states (^1^A^RKS^) are found at higher energies of 0.321 and 0.253 eV, respectively. Using the TPSSh functional, similar results are obtained. Again, the triplet states are found as ground states, while broken-symmetry states are slightly higher in energy for both complexes. The broken-symmetry states have slightly higher energies of 0.037 and 0.041 eV, respectively, compared to the B3LYP functional. Moreover, at energies of 0.197 and 0.146 eV, respectively, closed-shell singlet states are found, which are moderately lower than the B3LYP functional results. All the broken-symmetry states have values of ⟨*S*^2^⟩ close to 1, which attests that they are approximately equal weight mixtures of singlet and triplet states, and the small energy separation from the triplet terms shows that there is only a weak coupling between the two unpaired electrons. For the broken-symmetry states of both complexes, the spin populations on the Cu atoms amount to values close to 0 (despite the large values for ⟨*S*^2^⟩), which indicates that the unpaired electrons are essentially not located at the Cu atoms. Thus, the low-lying electronic states do not correspond to states with one electron located on the ligands and the other at the Cu atom. Visualizing the molecular orbitals reveals a similar result (see Supporting Information, Figures S3, S5, and S6).

**Table 2:** Relative Energies and Expectation Values of *S*^2^ of Different Low-Lying States of [Cu(L1)_2_]^+^ and [Cu(L2)_2_]^+^ by Density Functional Calculations with the B3LYP and TPSSh Functionals and the def2-TZVP Basis Set (including the D3 Dispersion Correction) and the Mulliken Spin Populations (SP) on the Copper atom^a^

		B3LYP + D3				TPSSh + D3			
complex	state	E/eV	⟨*S*^2^⟩	J/cm^–^^1^	SP(Cu)	E/eV	⟨*S*^2^⟩	J/cm^–^^1^	SP (Cu)
[Cu(L1)_2_]^+^	^3^A	0	2.02		0.16	0	2.02		0.26
	^BS^A	0.012	1.00	131	0.00	0.037	0.97	280	0.00
	^1^A^RKS^	0.321	0		0	0.197	0		0
[Cu(L2)_2_]^+^	^3^A	0	2.03		0.22	0	2.02		0.28
	^BS^A	0.023	0.97	243	0.00	0.041	0.95	313	0.00
	^1^A^RKS^	0.253	0		0	0.146	0		0

a ^BS^A denotes the broken-symmetry state, ^1^A^RKS^ denotes the closed-shell singlet state by restricted Kohn–Sham calculations. Additionally, the table presents the coupling constants *J* of the Heisenberg-Dirac-van-Vleck Hamiltonian , representing the singlet triplet splitting and was performed in the broken-symmetry geometry. The values are obtained according to the formula of Soda et al.^38^

Further functionals (e.g., CAM-B3LYP) do not yield qualitatively different results (see Supporting Information, Table S1). Additionally, a consideration of relativistic contributions, which often become important when investigating transition metal complexes^39,40^ can be found in the Supporting Information, Table S2. However, no significant deviations were found.

In order to investigate the redistribution of electron density from the ligands to the metal upon oxidation (RIET process), the Fukui functions^41^ for the three neutral complexes [Cu(L1)_2_], [Cu(L2)_2_], and [Zn(L1)_2_] were computed. The Fukui functions [*f*^–^(**r**)] were determined using finite differences between the electron densities of the neutral complexes at the ground state structures and the electron densities of the cations (triplet states) at the structures of the neutral complexes.^41,42^Figure 4 illustrates the Fukui functions for all three complexes. The plots of *f*^–^(**r**) show local regions distributed over the whole complex where the electron density is increased [negative value of *f*^–^(**r**)] and regions where the density is decreased [positive values of *f*^–^(**r**)]. In particular, the electron density is increased at the copper atom and decreased at the ligand atoms surrounding the metal atom. This phenomenon becomes clearly visible when comparing the *f*^–^(**r**) functions of [Cu(L1/L2)_2_] with [Zn(L1)_2_]. Upon oxidation of the latter, no increase of electron density at the metal is observed. On the other hand, the Fukui functions indicate RIET in both copper complexes, with no significant differences between [Cu(L1)_2_] and [Cu(L2)_2_].

To confirm that the density functional electronic structure is in accordance with the experimental UV–vis spectra, the absorption spectra for the triplet states were calculated using TDDFT (see Figure 5). For both complexes, the experimental absorption spectra are similar. There are sharp peaks at 230 nm and in the range of 314–362 nm, and there is a broader band at around 500 nm with a long tail toward higher wavelengths. Furthermore, for [Cu(L2)_2_]^+^, there is a shoulder at around 725 nm. In the case of [Cu(L1)_2_]^+^, this shoulder is absent, but the absorption around 1000 nm is slightly stronger. Finally, at long wavelengths of about 1900 nm, there is a very broad absorption that is considerably stronger for [Cu(L2)_2_]^+^ than for [Cu(L1)_2_]^+^. The large width of this band may be due to vibronic effects. The TDDFT spectra qualitatively agree with the experimental results. The calculated spectra exhibit a manifold of weak peaks at short wavelengths (≥295 nm) and strong peaks in the range of 304–345 nm, similar to the experimental spectra. Between 409 and 580 nm, both TDDFT spectra display three prominent peaks, followed by a weaker peak at about 730 nm. The long-wavelength region (experimental ≃1900 nm) corresponds to ligand-to-metal charge transfer, metal-to-ligand charge transfer (MLCT), or intervalence charge transfer (IVCT) processes.^43^ The calculated spectra indicate MLCT transitions at 1576 nm ([Cu(L2)_2_]^+^) and 1638 nm ([Cu(L1)_2_]^+^), respectively. However, it is known that TDDFT often overestimates the excitation energies of charge-transfer transitions compared to experiments.^43−45^ Nevertheless, the calculated intensities of these charge-transfer bands agree well with the experiment, as the intensity of the [Cu(L1)_2_]^+^ transition is noticeably weaker than that of [Cu(L2)_2_]^+^. Thus, there is a satisfactory agreement between the TDDFT calculations and the UV–vis measurements.

In summary, the density functional calculations do not find major differences between the electronic structures of the two complexes, in contrast to the experiments. The density functional calculations yield triplet ground states and no significant difference between the singlet–triplet splitting of the two complexes, whereas the magnetic measurements and NMR spectra indicate diamagnetic ground states and show that the magnitude of the singlet–triplet splitting clearly differs between the two complexes [Cu(L1)_2_]^+^ and [Cu(L2)_2_]^+^. Moreover, the density functional calculations find symmetric structures for both complexes, whereas in the case of [Cu(L2)_2_]^+^, the X-ray diffraction measurements indicate differences in the structures of the two ligands. Finally, concerning the spin density at the copper atom, the density functional calculations do not predict a pronounced difference between the two complexes (see Table 2), whereas the EPR measurements point to a nonvanishing spin density at the copper atom of [Cu(L2)_2_]^+^ in contrast to [Cu(L1)_2_]^+^. Thus, the density functional calculations do not satisfactorily explain the experimental results. Apparently, in the present scenario, Kohn–Sham density functional methods fail to accurately describe the electronic structure. Hence, it is advised to also consider multiconfigurational methods.

### 2.3 Multiconfigurational Methods—Selection of Active Space

The experiments, which indicate two unpaired electrons and a singlet ground state, as well as the density functional calculations, which yield very low-lying broken-symmetry states, clearly point to a multiconfigurational character of the complexes. Consequently, the identification of the key molecular orbitals is critical to ensuring their inclusion within the active space.

The construction of an active space capable of incorporating all crucial electronic states demands meticulous consideration of the experimental findings as well as the output of DFT calculations. It is reasonable to hypothesize that the electronic structures of the complexes [Cu(L1)_2_]^+^ and [Cu(L2)_2_]^+^ in a solution environment exhibit symmetry, given the absence of any compelling reason for an unpaired electron to preferentially occupy one of the chemically equivalent ligands. However, this situation may shift in the solid state due to packing effects, as alluded to by the experimental results.

As indicated by the experiments, it is clear that the unpaired electrons in principle may reside on the Cu atom as well as on both of the ligands; thus, the corresponding orbitals should be included in the active space. This is also indicated by the structures of Figure 1b.

**Figure 6:**
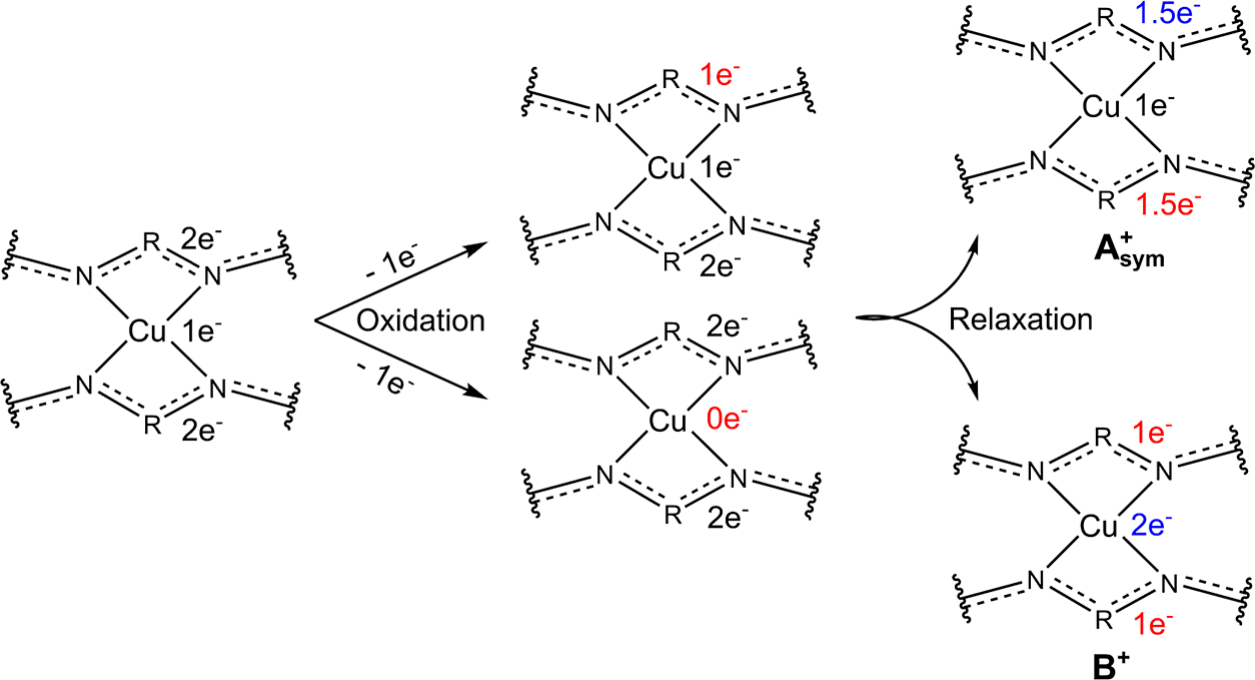
Schematic mechanism for one-electron oxidation of [Cu(L1/L2)_2_], assuming that the unpaired electron is located on the copper atom before oxidation for the sake of simplicity. The two different intermediate states that may be formed upon oxidation undergo electron relaxation to adopt the symmetric structures of the monocationic complexes.

A Gedankenexperiment is helpful to find such an active space. Figure 6 illustrates the possible oxidation processes of the neutral complex, which has a doublet multiplicity (spin = 1/2) due to one unpaired electron. According to the density functional calculations on the neutral complexes (Table 2), about 40% of the spin density resides on the copper atom, and the largest part of the remaining spin density is located on the nitrogen atoms adjacent to the copper atom. Thus, for the sake of simplicity, in Figure 6, it is assumed that the single electron is located on the copper atom, as confirmed by EPR spectroscopy (see Supporting Information, Figure S1). The removal of one electron from the neutral complexes could be realized in different ways. First, it may be abstracted from one of the ligands, leading to an asymmetric species where 1 e^–^ is on one ligand whereas the other ligand still has 2 e^–^. Assuming that in the gas phase no asymmetric form appears, this electronic structure should relax into one of two possible symmetric structures. Alternatively, an electron may be extracted from the copper atom, leading to a symmetric, diamagnetic structure with a formal Cu(III) atom. However, a Cu(III) oxidation state is unlikely,^46^ especially in the presence of the electron-donating ligands, and is also not compatible with the experimental results. As the experiments clearly indicate a paramagnetic character, this structure is likely to relax into one of the electronic structures of the first scenario. The bottom structure in Figure 6 reflects the electronic state found in the density functional calculations and postulated in the preceding study as structure **B**^**+**^ (Figure 1b). In contrast, the top structure, which will be called **A**_sym_^+^, also has a symmetric character, but only 1 e^–^ is located at the copper atom and 1.5 e^–^ on each ligand. Here, a Cu(II) atom is present while a symmetric structure is preserved.

Another aspect that confirms the active space is the nature of the molecular orbitals of the neutral complexes [Cu(L1)_2_] and [Cu(L2)_2_], which can be described by single configurations. In the Supporting Information, section 6 a brief discussion of the energetic properties of the molecular orbitals for [Cu(L1)_2_] and [Cu(L2)_2_] can be found. Based on these analyses, the MOs 149–151 certainly are crucial for the description of the low-lying electronic states of the [Cu(L1)_2_]^+^ and [Cu(L2)_2_]^+^ complexes. The consideration of these orbitals leads to a minimum active space of 4 electrons (one electron is removed from the neutral complexes) in 3 orbitals, CAS(4,3), for the monocationic complexes [Cu(L1)_2_]^+^ and [Cu(L2)_2_]^+^.

One configuration is expected to describe state **B**^**+**^ and is denoted [211]. At this point, we introduce a new notation: The bracket term stands for an electronic configuration, and the first digit represents the occupation of a mainly copper molecular orbital and the latter two of two delocalized ligand orbitals. Then there are two different configurations, [121] and [112], that both correspond to states belonging to the distribution **A**_sym_^+^ with 1.5 e^–^ on each ligand.

It is crucial to preserve the symmetry of the active space. To ensure the flexibility of the active space during the investigation of the potential energy surfaces (PES), correlating counterparts of the discussed orbitals are included. This leads to an active space of 4 electrons in 6 orbitals, CAS(4,6), which is chosen for further investigation of the electronic structure of the monocationic complexes [Cu(L1)_2_]^+^ and [Cu(L2)_2_]^+^.

**Figure 7:**
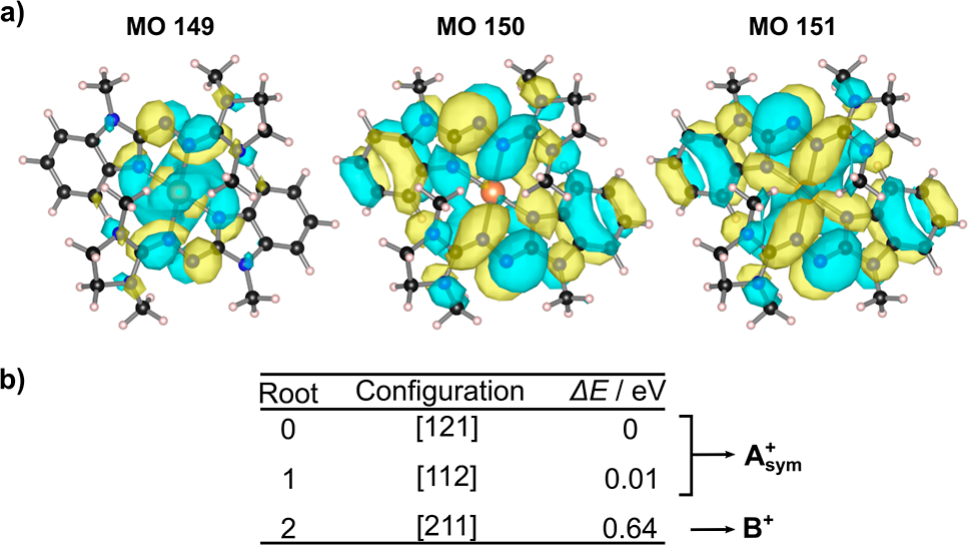
(a) Plots of the isodensity surfaces for the three strongly occupied MOs of [Cu(L1)_2_]^+^ from a state-averaged CASSCF (4,6) calculation at the neutral complex structure (B3LYP/def2-SVP). Three roots were included with a weight of 0.25, 0.25, and 0.50. (b) Leading configurations of the included roots and their energy differences Δ*E*. Roots 0 and 1 correspond to an electronic structure that can be attributed to **A**_sym_^+^, whereas root 2 can be attributed to **B**^**+**^ in Figure 1b.

Figure 7 illustrates the occupied molecular orbitals of a preliminary state-averaged (SA) CASSCF (4,6) calculation for [Cu(L1)_2_]^+^, including the three lowest-lying roots of singlet multiplicity (weight: 0.25, 0.25, and 0.50). It can be seen that the two MOs 150 and 151 are two combinations of ligand orbitals, with MO 151 having a copper contribution, whereas MO 149 is clearly stronger localized at the copper atom. Comparing the energies of the different roots, it becomes clear that two roots (0 and 1) are quasi-degenerate (Δ*E* = 0.01 eV) and correspond to an electronic structure with an average occupation of about 1.5 e^–^ on each ligand, while 1 e^–^ is located at the Cu MO 149 in accordance with the structure **A**_sym_^+^ of the Gedankenexperiment in Figure 6. On the other hand, the electronic structure of root 2 represents the state **B**^**+**^ (denoted [211]) that was suggested earlier^27^ and corresponds to the density functional ground state. In the latter case, both ligand MOs (150 and 151) are singly occupied, while the Cu MO 149 hosts two electrons. Consequently, the spin density is located almost entirely on the ligands, in agreement with the experimental observations for [Cu(L1)_2_]^+^. In the case of [Cu(L2)_2_]^+^, an electronic structure similar to that of [Cu(L1)_2_]^+^ is found (see Supporting Information, Tables S7 and S8.)

To confirm the validity of the relatively small active space of 4 electrons in 6 orbitals, CASSCF and NEVPT2 calculations with different larger active spaces have been performed (see Supporting Information, Table S10). It is found that the larger active spaces show no large differences compared to the CAS(4,6) active space. Also, larger basis sets have been employed, and only minor deviations are found (see Supporting Information, Table S9). Besides that, relativistic contributions using ZORA were considered, but no significant differences to the nonrelativistic treatment were found in terms of energy differences (see Supporting Information, Table S11).

In the next section, we discuss the energetic properties and the bond parameters for the identified electronic states.

### 2.4 Energy and Bond Properties of the Low-Lying Electronic States

At first, structure optimizations by CASSCF calculations using the CAS(4,6) space were performed for the three lowest-lying singlet states ([121], [112], and [211]) of the complexes [Cu(L1)_2_]^+^ and [Cu(L2)_2_]^+^. Although the density functional calculations yield triplet ground states, singlet states were considered here, as experimental evidence points to singlet ground states. However, since there are low-lying triplet states and, in parts, the calculations yield triplet ground states, the subsequent calculations will also include the triplet states. A detailed discussion of the CASSCF structure optimization can be found in the Supporting Information, Section 10. In accordance with expectations, the CASSCF structures reveal copper ligand bond distances that differ significantly from the crystal structure (see Supporting Information, Table S6). Especially, the N^3^–Cu bond distances of [Cu(L1)_2_]^+^ and [Cu(L2)_2_]^+^ ([211] configuration) deviate by 0.622 and 0.485 Å (2.717 vs 2.095 Å and 2.471 vs 1.962/1.986 Å). Furthermore, the inclusion of dynamic correlation leads to significant changes in the relative energies of the three states (see Supporting Information, Table S13), revealing that the CASSCF structure is far from the optimal structure due to the lack of dynamic correlation.

Unfortunately, analytic gradients for multireference methods are nontrivial, rarely available, and resource-demanding, and numerical gradients are also not viable for molecules of about 70 atoms using a CAS(4,6) reference space and a multireference correlation treatment. However, the CASSCF (4,6) structure optimization indicated that the most significant deviations in the bond parameters occur around the copper atom, whereas the other bond parameters of the ligands in the state-optimized structures are more similar (see Supporting Information, Table S6). Thus, the distances of the ligands to the copper atom presumably are in particular responsible for the significant energy differences between the electronic states. This can be explained by the different bonding situations in the three electronic states: In the cases of [121] and [112] states, the two unpaired electrons are shared by the ligands and the copper atom, while in [211], the two unpaired electrons are located exclusively on the ligands.

**Figure 8:**
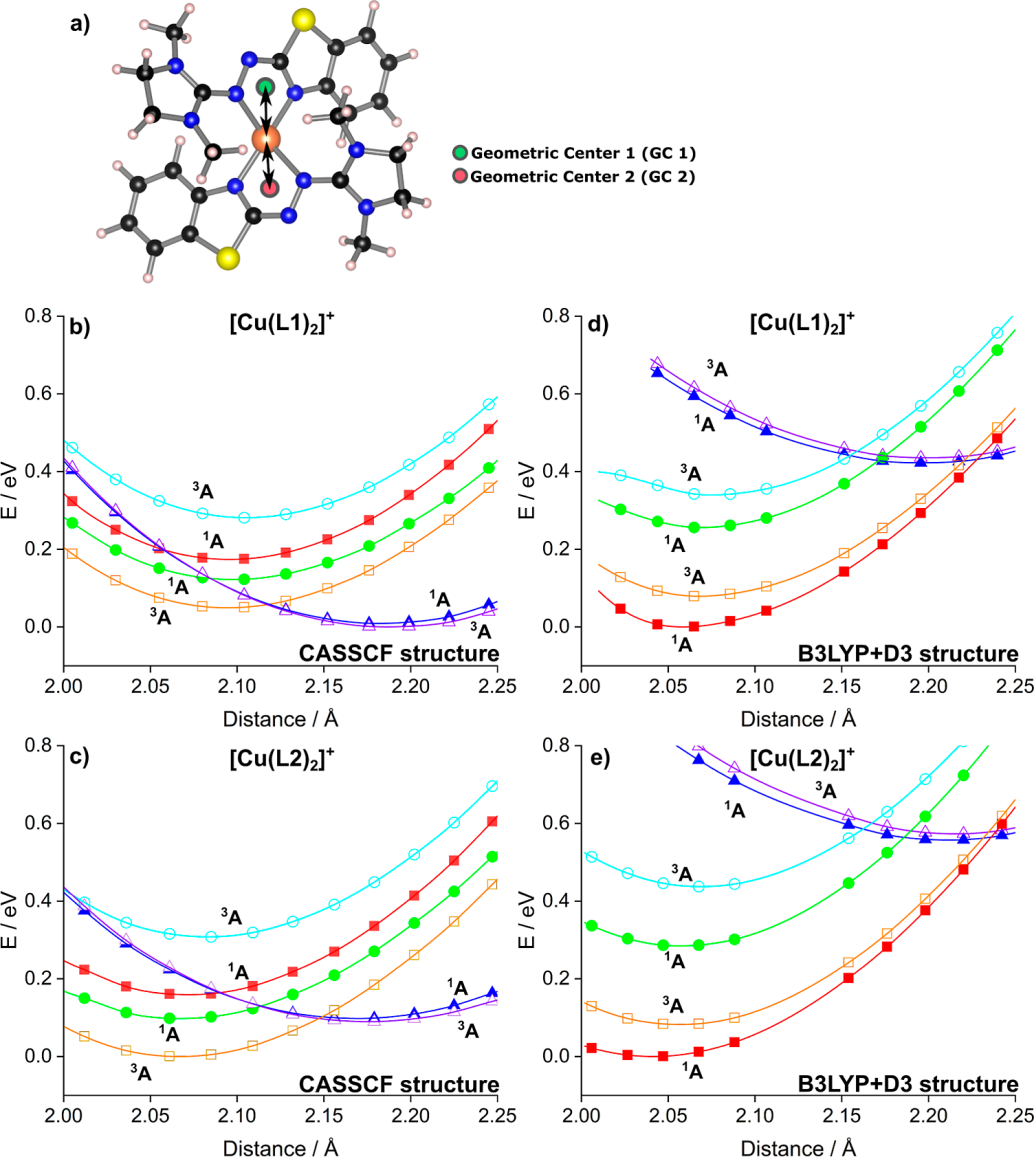
NEVPT2-CASSCF/def2-TZVPP 1D-scans (symbols) and fitted curves (lines) for two multiplicities (^1^A (*S* = 0), ^3^A (*S* = 1), weighting of multiplicities: 0.5 and 0.5) and three roots (weighting of roots: 0.25, 0.25, and 0.5), respectively. Squares: [121] states, circles: [112] states, and triangles: [211] states; filled symbols: ^1^A states and open symbols: ^3^A states. Two different geometrical structures were used for each oxidized complex. (a) Schematic representation of scans along the geometric centers 1 (GC 1) and 2 (GC 2). In the following plots, both centers were scanned at an equal distance with respect to the copper center (leading to a 1D scan). More information about the potential surfaces (2D) can be found in Supporting Information, section S11. (b) 1D scan of all six roots of [Cu(L1)_2_]^+^ relying on the CASSCF (4,6)/def2-SVP optimized structure. (c) 1D scan of all six roots of [Cu(L1)_2_]^+^ relying on the B3LYP + D3/def2-TZVP optimized structure. (d) 1D scan of all six roots of [Cu(L2)_2_]^+^ relying on the CASSCF (4,6)/def2-SVP optimized structure. (e) 1D scan of all six roots of [Cu(L2)_2_]^+^ relying on the B3LYP + D3/def2-TZVP optimized structure.

Based on this reasoning, it was decided to approximately search for the minima of the different electronic states using a method including dynamical electron correlation by scanning the distance between the copper atom and the ligands with otherwise frozen structures. For this purpose, the structures of a state-averaged CASSCF calculation (0.25 [121], 0.25 [112], and 0.5 [211] ^1^A states) as well as the B3LYP/def2-TZVP structures were chosen as starting structures. For each ligand, a reference point was defined by using the centroid of the four atoms (C^1^, N^2^, N^3^, and C^2^) that are close to the copper atom. Figure 8a illustrates the two centroids for [Cu(L1)_2_]^+^. Then, points on the PESs were generated by shifting the two frozen ligands along the direction from the copper atom to the centroid. Plots of the two-dimensional (2D) PESs are shown in Figure S12 of the Supporting Information. The resulting two-dimensional PESs are symmetrical with respect to the two Cu–ligand distances. Therefore, cuts for the diagonal points (the same Cu–L distance for both ligands) are shown in Figure 8b–e. These scans also include the triplet states. Moreover, a larger basis set (def2-TZVPP) was used, which is recommended when using dynamic correlation methods.

For [Cu(L1)_2_]^+^, the lowest-lying state by the scans using the CASSCF structure is the [211] ^3^A state with a Cu–centroid distance of 2.187 Å, the [211] ^1^A state being higher by only 0.01 eV (Figure 8b). The [121] ^3^A, [112] ^1^A, [121] ^1^A, and [112] ^3^A states are found at 0.05, 0.12, 0.17, and 0.28 eV, respectively. By contrast, for [Cu(L2)_2_]^+^, the lowest-lying state by the scans using the CASSCF structure is the [121] ^3^A state with a Cu–centroid distance of 2.067 Å (Figure 8c). The [211] ^3^A and ^1^A states are found slightly higher by 0.09 and 0.10 eV, and the [112] ^1^A, [121] ^1^A, and [112] ^3^A states have relative energies of 0.10, 0.16, and 0.31 eV, respectively. Thus, there is a significant difference from the results at the CASSCF structures (Table S13), where ^1^A [112] and [121] are 1.36 and 1.03 eV higher in energy compared to ^1^A [211], respectively. In particular, the [121] and [112] states are lowered considerably compared to the energy separations at the CASSCF structures. Thus, by allowing certain structural relaxation when including dynamical correlation, the energetic difference between the states belonging to the three different configurations is small again for both complexes, similar to the CASSCF (4,6) results in Table S13.

To summarize, according to the two-dimensional scans relying on the CASSCF structures, the [Cu(L1)_2_]^+^ complex prefers the [211] configuration. The differences between the low-lying electronic states of both complexes are, however, small, indicating possible transitions between these states, even at lower temperatures.

It is questionable, however, if the structures obtained by the CASSCF calculations form a good basis for calculations including dynamical correlation both for the single point calculations and for the scans of the Cu–ligand distances, also in view of the large differences between the CASSCF structures and the crystal structures. Therefore, additionally, the B3LYP + D3/def2-TZVP ground state structures were used to investigate the energy separations of the different electronic states using NEVPT2-CASSCF (Figure 8d,e). Also, de Bruin et al., in their NEVPT2-CASSCF investigations of redox-active Co complexes, relied on density functional structures.^47−49^ Moreover, since the crystal structures presumably are the most reliable structures at hand, the different energy separations were also determined at the experimental crystal structures.

Table 3 shows the relative energies of the six lowest-lying electronic states at the B3LYP + D3 structures of the triplet states. These calculations yield a qualitatively different order of the electronic states. For both complexes, the lowest-lying state is the [112] ^1^A state, and close-by, at about 0.04 eV, there is the [121] ^3^A term. For the [Cu(L1)_2_]^+^ complex, the [211] ^1^A and ^3^A terms with doubly occupied Cu 3d orbitals are close in energy, at 0.13 and 0.14 eV, whereas for the [Cu(L2)_2_]^+^ complex, the [211] terms are markedly higher in energy, at about 0.4 eV. Figure 8d,e shows the results of the scans of the two Cu–L distances with frozen ligands, relying on the density functional structures. In contrast to the CASSCF structure scans, for both complexes, the lowest-lying state is the [112] ^1^A state with short Cu–centroid distances of 2.059 ([Cu(L1)_2_]^+^) and 2.041 ([Cu(L2)_2_]^+^) Å, and the lowest-lying triplet term is the [121] ^3^A term at 0.08 eV. Within the scans, the ^1^A [211] states are found at energies higher by 0.42 eV ([Cu(L1)_2_]^+^, 2.196 Å) and 0.56 eV ([Cu(L2)_2_]^+^, 2.211 Å). The differences in the Cu–L distances between the [211] states on the one hand and the [121] and [112] states on the other hand nicely reflect the different occupations of the Cu 3d orbitals. Whereas for the [Cu(L2)_2_]^+^ complex, both the CASSCF and the B3LYP + D3 structure scans are in line in terms of which type of electronic structure is more preferable (the Cu(II)-like), for [Cu(L1)_2_]^+^, the character of the electronic ground states found by the two scans differs. However, in both scenarios, the energy of the [211] states of [Cu(L1)_2_]^+^ with respect to the [121] and [112] states is lower than that of the corresponding states of [Cu(L2)_2_]^+^, indicating a better stabilization of the [211] state for the L1 ligand system.

**Table 3:** Relative Energies of the Lower-Lying Electronic States of [Cu(L1)_2_]^+^ and [Cu(L2)_2_]^+^ by NEVPT2-CASSCF (4,6) Calculations with the def2-TZVPP Basis Set at the Corresponding B3LYP + D3/def2-TZVP Structures for the ^3^A States and at the Experimental Crystal Structures

		B3LYP + D3 structure		crystal structure^a^	
complex	state	Δ*E*^CASSCF^/eV	Δ*E*^NEVPT2^/eV	Δ*E*^CASSCF^/eV	Δ*E*^NEVPT2^/eV
[Cu(L1)_2_]^+^	[112] ^1^A	0	0	0.001	0.162
	[121] ^3^A	0.013	0.036	0	0.160
	[121] ^1^A	0.116	0.221	0.103	0.358
	[112] ^3^A	0.142	0.276	0.155	0.471
	[211] ^1^A	1.557	0.128	1.911	0
	[211] ^3^A	1.557	0.141	1.914	0.020
[Cu(L2)_2_]^+^	[112] ^1^A	0	0	0	0
	[121] ^3^A	0.000	0.040	0.116	0.228
	[121] ^1^A	0.120	0.244	0.499	0.465
	[112] ^3^A	0.188	0.359	0.564	0.601
	[211] ^1^A	2.069	0.390	3.184	1.045
	[211] ^3^A	2.067	0.413	3.188	1.103

a Hydrogen atoms are optimized.

Finally, the calculations based on the crystal structures (with the positions of the hydrogen atoms optimized) yield interesting differences between the two complexes. For the [Cu(L2)_2_]^+^ complex, the same ground state as at the B3LYP + D3 structure is found: The [112] ^1^A state is the most preferred one, but the low-lying [121] ^3^A term already has an energy of 0.23 eV, and the two [211] states are much higher in energy (≃1 eV). In contrast, for the [Cu(L1)_2_]^+^ complex, similar to the results at the CASSCF structure, the [211] states are the preferred ones, the [211] ^1^A state being the most stable and the [211] ^3^A term being higher in energy by only 0.02 eV, and also the lowest-lying [112] and [121] states are not far in energy (the lowest excitation energy being 0.16 eV).

Considering all calculations from Figure 8 and Table 3, it reveals that the [Cu(L2)_2_]^+^ complex is most stable in a Cu(II)-like configuration (corresponding to **A**_sym_^+^), whereas the [Cu(L1)_2_]^+^ complex seems to be more favored in the Cu(I)-like configuration (corresponding to **B**^**+**^). In relation to that, the previous study of the [Cu(L1/L2)_2_]^+^ complexes^27^ postulated a substantial contribution of the **C**^**+**^ configuration. This cannot be confirmed by the present CASSCF and NEVPT2 calculations. The relative energy of the Cu(I)-like [211] states with respect to the [121] and [112] states is significantly higher for the L2 complex compared to the L1 complex, indicating a ligand-dependent stabilization of the two electronic structures. Therefore, in the next section, we will explore the change of the relative energies of the electronic states with the alteration of the ligand systems.

### 2.5 Ligand Tuning

As we continued our investigation, we sought to explain the differences in stability between the electronic states of various ligand systems. One crucial factor is the electron donor and acceptor behavior of the ligands, the electron-donor strength originating from the stabilization of the oxidized ligand state through π-conjugation. For ligand L1, the NCH_3_ group’s N atom is sp^2^-hybridized, and its filled p-orbital integrates directly into the π-system. In contrast, ligand L2’s sulfur atom’s lone pairs are less engaged in π-bonding (see Table S6). This π-destabilization in L2 (S) relative to L1 (NCH_3_ group) leads to a reduced electron donation to the copper atom, preserving a more stable π-system. The higher *E*_ox_ value in the cyclic voltammetry measurements for HL2 compared to HL1 emphasizes this mesomeric effect (Figure 2a).

On the other hand, the inductive effect due to the differences in electronegativity between the NCH_3_ group and the S atom must be considered. S (L2), having a lower electronegativity, allows for more electron donation to the copper atom, supporting a [211] configuration. Conversely, the NCH_3_ group (L1) with a higher electronegativity binds electrons more tightly, favoring the [121] and [112] states. However, this argument contradicts the results presented in Figure 8 and Table 3. The lower electronegativity of S (L2) is expected to stabilize the [211] state by enabling the ligand to share more electron density with the copper atom. However, for the considered structures, the energies of the [211] states with respect to the [121] and [112] states are less high in the case of [Cu(L1)_2_]^+^ than in the case of [Cu(L2)_2_]^+^. Therefore, electronegativity might not play a key role in stabilizing these electronic structures.

**Figure 9:**
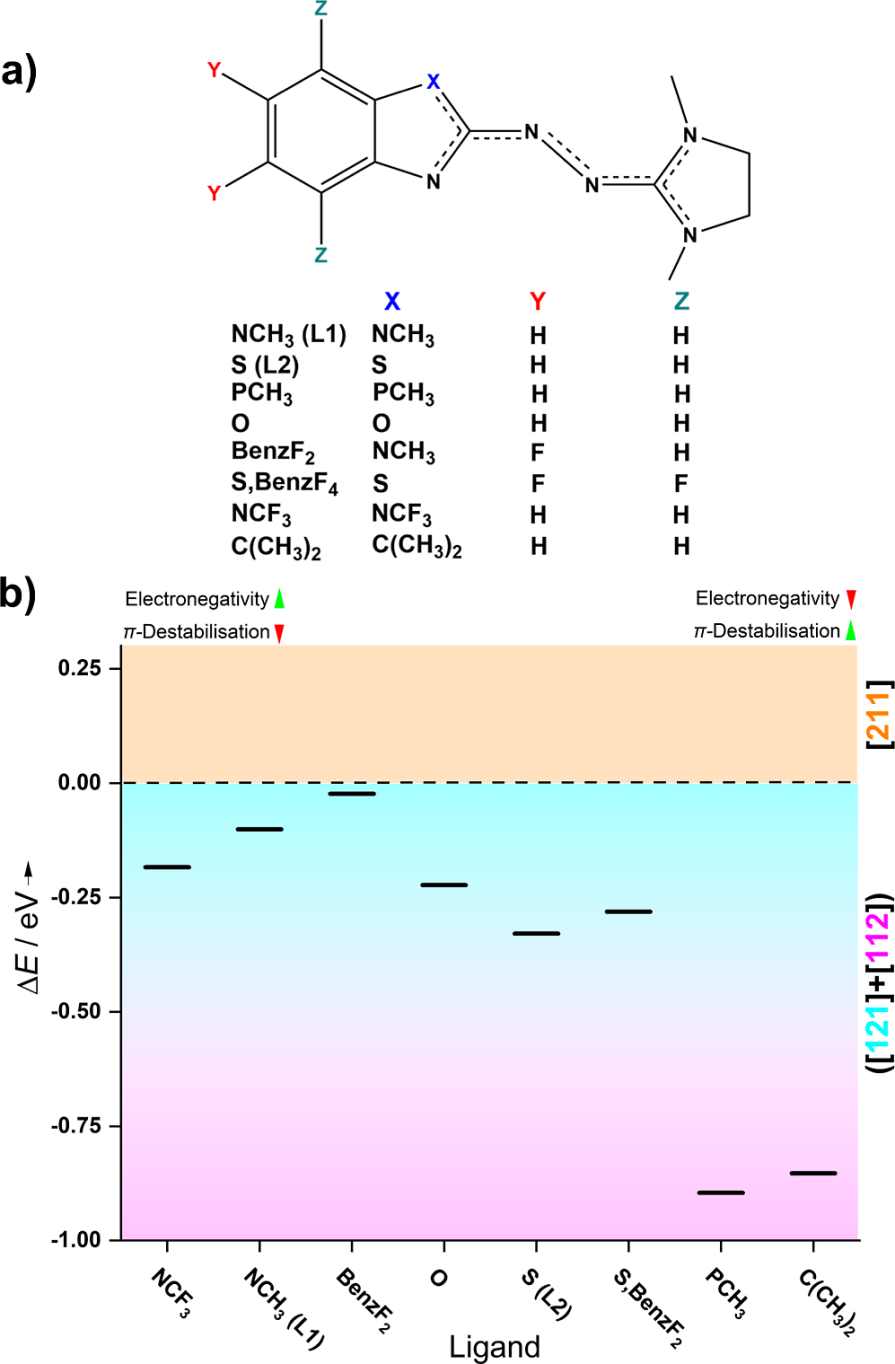
(a) Schematic representation of different ligands’ functional groups. Details are in the Supporting Information, Figure S13. (b) Energy differences (NEVPT2-CASSCF (4,6)/def2-TZVP) between the [121] or [112] state and the [211] state for various copper complexes at the B3LYP + D3/def2-TZVP structure, sorted by electronegativity and π-destabilization. Green arrows indicate a strong effect; red arrows, a weak effect. Energies for the respective scans in the SA-CASSCF structure are in Figure S14 in section S13.

To validate both effects (π-stabilization and inductive), we performed further calculations with modified ligands, introducing additional functional groups with varied electronegativities and π-destabilization behaviors (see Figure 9a). We chose six additional ligands and evaluated the relative energies of the six electronic states (see Supporting Information, Figure S13). These ligands represent two kinds with opposite properties: the first group exhibits high electronegativity and low π-destabilization (Figure 9b, left side), while the second shows strong π-destabilization but lower electronegativity (Figure 9b, ride side). We relied on the B3LYP + D3/def2-TZVP structures and extracted the energy differences Δ*E* between the different electronic states. However, scans of the NEVPT2-CASSCF PESs for all copper complexes relying on a CASSCF structure were also performed, and energy differences can be found in Figure S14.

Figure 9b illustrates energy differences between the lowest-lying of the [121] or [112] states and the lowest-lying [211] state for the monocationic copper complexes. All complexes prefer either the [121] or the [112] state, similar to the results of Table 3. However, the ligands with a lower π-destabilization (left side) favor the [211] structure in comparison to the other ligands (right side), aided by the lone pairs at the X position (Figure 9b). For X = NR, the +M effect is maximized. Substituting the NR group with O increases the π-destabilization, destabilizing the [211] state. In general, ligands with strong π-destabilization (right side of Figure 9b) prefer the [121] and [112] states with lower electron density on the copper atom, induced by groups at the X position that do not contribute to the π-system. Thus, the mesomeric effect appears to dominate the stabilization of the respective electronic structures.

In the cases of the ligands with PCH_3_ and O, the counterparts of L1 and L2, in terms of electronegativity, the calculations reveal that the observed behavior is essentially not due to the inductive effect. For example, PCH_3_, with much lower electronegativity than NCH_3_ (L1), should stabilize [211] if the inductive effect was decisive. However, for the PCH_3_ ligand, similar to L2, the [121] and [112] states are more favored, which can be linked to the destabilization of the π-system. This destabilization is further indicated by the bond angles between the ligand plane and the CH_3_ group, with ∠(plane-P-CH_3_) = 109.8°. Introducing a C(CH_3_)_2_ group instead of an NCH_3_ group shows a trend similar to the PCH_3_ group. Here again, the sp^3^-hybridization of the carbon atom within the aromatic system leads to a destabilization of the π-system. The C(CH_3_)_2_ group, unlike the PCH_3_ group, is a promising candidate for laboratory synthesis and could be further investigated spectroscopically.

The oxygen analogue of the L2 ligand with large electronegativity slightly stabilizes the [211] states, similar to L1. This is contrary to what would be expected if the inductive effect was a significant factor in stabilizing the respective electronic states. Furthermore, when introducing electron-drawing fluorine atoms to the system (BenF_2_, S,BenzF_4_, and NCF_3_), no significant stabilization effect of the [121] or [112] states is found compared to their fluorine-free analogues (see also CASSCF structure scans in Figure S14b). For example, the S,BenzF_2_ complex shows an energetic behavior similar to the L2 complex (S) (−0.028 eV), even though the [211] state is slightly more favored. For the ligand with the strongly electron-drawing NCF_3_ group, a minor stabilization of the [121] and [112] states is found at the B3LYP + D3 structure (−0.18 eV). Despite the fluorine groups’ drastic inductive effect, no significant energy deviation compared to their nonfluorine counterparts is observed.

In conclusion, the contribution of a functional group to the π-system appears to be the most crucial factor in stabilizing a particular electronic structure of the complexes. According to the calculations, electronegativity plays a relatively subordinate role.

### 2.6 Spin-Dependent Properties

Now that the electronic structure is understood, it is possible to examine the divergent behavior of the magnetic susceptibility of the two monocationic complexes in the solid state. As shown in the experimental overview (Figure 2), not only are the bond parameters of the two complexes different ([Cu(L2)_2_]^+^ exhibits a slightly asymmetric structure with two different ligands), but they also differ in their paramagnetic behavior: [Cu(L1)_2_]^+^ is characterized by a stronger paramagnetism beginning already at lower temperatures, whereas [Cu(L2)_2_]^+^ shows more of a diamagnetic behavior (see the SQUID curves in Figure 2b).

This result clearly differs from studies on Cu(II) complexes with two iminosemiquinolato ligands. In the latter case, a strong antiferromagnetic coupling was found between the unpaired electrons of the two ligands.^50^ It is also worth mentioning that the magnetic coupling in Cu(I) bis(verdazyl) complexes strongly depends on the coordination geometry, which in turn is influenced by crystal packing effects.^51−54^

**Figure 10:**
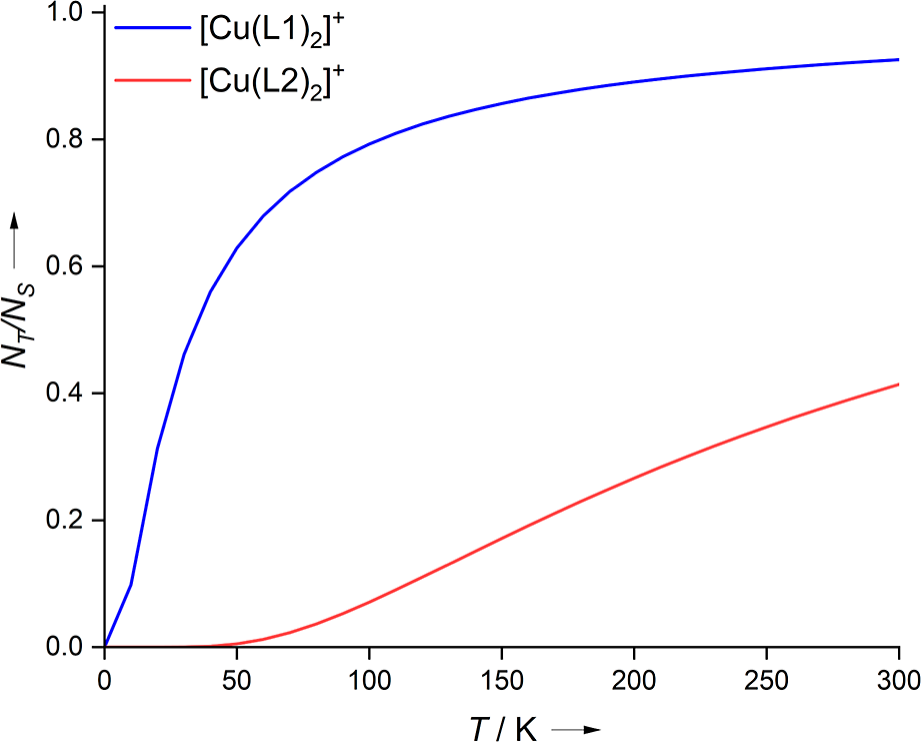
Calculated temperature-dependent population of the lowest-lying triplet states of the two Cu complexes [Cu(L1)_2_]^+^ and [Cu(L2)_2_]^+^ at their crystal_exp_ structures (*T* = 293.15 K, NEVPT2-CASSCF (4,6)/def2-TZVPP).

The calculated NEVPT2 energy differences between the lowest-lying singlet and triplet terms of [Cu(L1)_2_]^+^ and [Cu(L2)_2_]^+^ can now be used for an evaluation of the Boltzmann factor
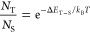
1where *N* is the population of the states, Δ*E*_T–S_ is the energy difference between the singlet and triplet states, *k*_B_ is the Boltzmann constant, and *T* is the temperature. For this evaluation, the results of the crystal structures are used, as they seem to be the most reliable ones. Figure 10 illustrates the temperature dependence of the population of the lowest-lying triplet states of the two complexes. For both complexes, the simulated curves nicely agree with the SQUID curves in Figure 2b. Thus, energy splittings obtained by the NEVPT2 calculations (at the crystal structures) are able to well reproduce the observed magnetometric curves, suggesting that the right description of the electronic states has been met. This also emphasizes that the structure of the complexes is crucial, including symmetry breaking that may be the result of crystal packing. In this context, it is interesting to realize that the triplet states in particular become unfavorable when the system is forced into an asymmetric configuration. An explanation for this phenomenon is nontrivial, as stated in earlier work^55^ and might arise through a complex combination of exchange, kinetic energy, nuclear attraction, electron repulsion, and orbital relaxation contributions in both the single-reference triplet and multireference open-shell singlet states.^55^

## 3 Conclusions

The mononuclear homoleptic copper complexes with two urea azine (L1) or two thio-urea azine (L2) ligands reveal a rich redox chemistry and an ambivalent behavior of the electronic structure after one-electron oxidation to the monocationic complexes. For both complexes in their monocationic redox states, the quantum chemical calculations find several low-lying electronic states possessing two unpaired electrons. The ground states of both complexes are open-shell singlet states, as has been concluded from the magnetometric and NMR experiments.

The NEVPT2-CASSCF studies, relying on the crystal structures, show that the [Cu(L1)_2_]^+^ complex has an electronic ground state with a leading configuration in which all Cu 3d orbitals are doubly occupied ([211], **B**^**+**^ like), corresponding to a Cu(I) atom. In contrast, the [Cu(L2)_2_]^+^ complex has a ground state with a leading configuration in which one of the Cu 3d orbitals is singly occupied ([112], **A**^**+**^ like), indicating the essential contributions of configurations with a Cu(II) atom. The finding of Cu(II) in [Cu(L2)_2_]^+^ corroborates the interpretation of the EPR spectra in terms of spin–orbit coupling, which for the L2 complex show a distinct shift of the *g* value with respect to the free electron value in contrast to the L1 complex. For [Cu(L1)_2_]^+^, the separation between the ground term and the lowest-lying triplet term of 0.02 eV is quite small, and there are also low-lying states with Cu(II) character. For [Cu(L2)_2_]^+^, in contrast, the gap between the ground term and the lowest-lying triplet term of 0.23 eV is already relatively large, and the separation to the lowest-lying Cu(I) states is still considerably larger. Thus, the different singlet–triplet splittings readily explain the magnetometric experimental findings at variable temperatures.

Considering the result that the Cu in [Cu(L1)_2_]^+^ can be assigned the oxidation number I, but in [Cu(L2)_2_]^+^ rather the oxidation number II, it can be stated that on the oxidation of [Cu(L1)_2_] there is RIET, but there is at most partial RIET on the oxidation of [Cu(L2)_2_].

The energetic stabilization of one of the electronic configurations is linked to the functional groups in the ligands. It could be shown that the varying contribution of the respective functional group to the π-system causes the electron density to be pulled toward or pushed away from the copper atom. This effect ultimately leads to the stabilization of one of the two electronic configurations. The rational approach followed in this work for the evaluation of the electronic structures is applicable to other complexes, thereby contributing to a better understanding of the properties and reactivities of complexes with redox-active ligands and metals. Nevertheless, the theoretical investigation of the real structures in solution or vacuum of such complexes remains challenging due to the present inability to determine the structures of larger systems with multireference correlation methods with reasonable resource demand.

## 4 Computational Methods

The density functional calculations as well as the wave function-based multireference calculations were carried out with the program package ORCA, version 5.0.3.^56^ The density functional calculations used the B3LYP functional together with the def2-TZVP basis set^57^ (unless otherwise noted). A validation of different basis set sizes and methods can be found in the Supporting Information, Table S1. The CASSCF and multireference calculations use the def2-SVP and def2-TZVPP basis sets.^57^

The orbitals for the multireference calculations were obtained by complete active space self-consistent field (CASSCF) calculations. The dynamic electron correlations was accounted for by different flavors of multireference perturbation theory, namely second-order n-electron valence state perturbation theory (NEVPT2), second-order complete active space perturbation theory (CASPT2), and the dynamic correlation dressed complete active space method [DCD-CAS(2)].^56^ The selection of the active space is described in detail in the results section. For single point calculations, the chain of spheres approximation for the exchange integrals (COSX) and the approximate resolution-of-the-identity for the coulomb integrals (RIJ) were used in combination with the appropriate def2/JK (for multireference calculations) auxiliary basis set.^58,59^ The CASSCF structure optimizations were carried out without the approximate resolution-of-the-identity (RI) due to an erratic behavior of the gradient when using RI in combination with CASSCF in ORCA.

In order to trace the migration of electrons during a RIET, so-called condensed Fukui functions^60^ were calculated. These functions measure the shift of the electron density in a molecule upon reduction or oxidation, and a RIET process is characterized by a negative value. The calculations of the Fukui functions were carried out as described in the literature.^41^ Experimental methods can be found in the Supporting Information, Section 1.

## Supporting Information Available

Experimental methods; analytical data: supporting ESR and ATR-IR spectra; energy differences by Hartree–Fock and density functional calculations with different functionals and basis sets; bond parameters and spin populations by density functional calculations with and without dispersion correction; absorption spectra by TDDFT calculations with B3LYP; molecular orbitals of neutral [Cu(L2)_2_]; bond parameters of the complexes [Cu(L1)_2_]^+^ and [Cu(L2)_2_]^+^ by CASSCF calculations; leading configurations in the CASSCF(4,6) wave functions of the [121], [112], and [211] states of the [Cu(L1)_2_]^+^ and [Cu(L2)_2_]^+^ complexes; validation of active space and basis set size; structure optimization with CASSCF and inclusion of dynamic correlation; 2D surfaces by CASSCF(4,6)/def2-SVP calculations; calculated structures of further [Cu(X)_2_]^+^ complexes; calculated energies of [Cu(X)_2_]^+^ complexes with further ligands; energies of the cationic complexes by multireference calculations; total energies of different states of [Cu(L1)_2_]^+^ and [Cu(L2)_2_]^+^ by CASSCF and NEVPT2 calculations; and coordinates of [Cu(L1)_2_] and [Cu(L2)_2_]” (PDF)
