# Supplementary material for: Peculiar Differences between Two Copper Complexes Containing Similar Redox-Active Ligands: Density Functional and Multiconfigurational Calculations
Source: Inorg Chem. 2023 Dec 29;63(2):961–75. doi: 10.1021/acs.inorgchem.3c02949 (PMC10792600; doi:10.1021/acs.inorgchem.3c02949)
Supplement: Supplementary file 1 — ic3c02949_si_001.pdf [file ic3c02949_si_001.pdf]

On the Peculiar Differences of Two Copper Complexes Containing Similar Redox-Active Ligands: Density functional and Multiconfigurational Calculations - Supporting Information

Luca Gerhards[1*], Marco Werr[2], Olaf Hübner[2], Ilia A. Solov'yov[1,3,4], and Hans-Jörg Himmel[2*]

[1]Department of Physics, Carl von Ossietzky Universität Oldenburg, Carl-von-Ossietzky Str. 9-11, 26129 Oldenburg, Germany
[2]Anorganisch-Chemisches Institut, Ruprecht-Karls-Universität Heidelberg, Im Neuenheimer Feld 270, 69120 Heidelberg, Germany
[3]Research Center for Neurosensory Science, Carl von Ossietzky Universität Oldenburg, 26111 Oldenburg, Germany
[4]Center for Nanoscale Dynamics (CENAD), Carl von Ossietzky Universität Oldenburg, Institut für Physik, Ammerländer Heerstr. 114-118, 26129 Oldenburg, Germany
[*]Corresponding authors: Luca Gerhards, luca.gerhards@uol.de, Hans-Jörg Himmel, hans-jorg.himmel@aci.uni-heidelberg.de

December 8, 2023

Contents

1 Experimental methods S3

2 Analytical Data: Supporting ESR and ATR-IR spectra S4

3 Energy differences by Hartree-Fock and density functional calculations with different functionals and basis sets S5

4 Bond parameters and spin populations by density functional calculations with and without dispersion correction S11

5 Absorption spectra by TDDFT calculations with the B3LYP functional S14

6 Molecular orbitals of neutral $[Cu(L2)_2]$ S15

7 Bond parameters of the complexes $[Cu(L1)_2]^+$ and $[Cu(L2)_2]^+$ by CASSCF calculations S17

8 Leading configurations in the CASSCF(4,6) wavefunctions of the [121], [112], and [211] states of the $[Cu(L1)_2]^+$ and $[Cu(L2)_2]^+$ complexes S18

9 Validation of active space and basis set size S21

10 Structure optimization with CASSCF and inclusion of dynamic correlation S27

11 2D surfaces by CASSCF(4,6)/def2-SVP calculations S29

12 Calculated structures of further $[Cu(X)_2]^+$ complexes S30

13 Calculated energies of $[Cu(X)_2]^+$ complexes with further ligands S31

14 Energies of the cationic complexes by multireference calculations S32

15 Total energies of different states of $[Cu(L1)_2]^+$ and $[Cu(L2)_2]^+$ by CASSCF and NEVPT2 calculations S33

16 Coordinates of $[Cu(L1)_2]$ and $[Cu(L2)_2]$ S34

1 Experimental methods

A full description of the experimental details, i.e. the synthesis and analysis discussed in the main section, has already been published and can be found in the literature.[1] The experimental details of the newly measured ESR, ATR-IR, and SQUID data are described below.

X-band EPR spectra were measured with a *Bruker Elexsys E500 EPR* with an *ER 4116DM CW* dual-mode resonator. As a temperature system, an *ER 4112HV-CF58nc In-Cavity Cryogen Free VT* is used. The samples of the solid-state measurements were measured in glass capillaries and prepared in the glovebox *MBRAUN LABmaster DP (MB-20-G)* (Ar atmosphere). For better comparison of the spectra dI/dB was plotted against g, which was calculated for each sample using the spectrometer frequency f and magnetic field B: $g = hf/\mu_B B$

IR spectra were measured in the glovebox *SylaTech Y05G* on an *Agilent Cary 630* spectrometer equipped with a diamond ATR unit.

The magnetic properties of the substances were investigated using a *Quantum Design MPMS-XL-5 (5T)* SQUID magnetometer. The dependence of the molar susceptibility χ_M on the temperature was measured in a temperature range of 2–280 K and a field of 50 mT. Samples were prepared in the glovebox *MBRAUN LABmaster DP (MB-20-G)* (Ar atmosphere), the powdered samples were pressed into a polycarbonate capsule. A plastic straw served as a sample holder for the capsules. The change of χT as a function of temperature was measured again for $[Cu(L2)_2]PF_6$, as it was found that the diamagnetic behaviour of the probe makes it difficult to lock the probe at low temperature, locking the probe in the magnetometer at high temperature is necessary to obtain reliable results. A correction of the diamagnetic portion of the sample was carried out using the Pascal constant.[2]

2 Analytical Data: Supporting ESR and ATR-IR spectra

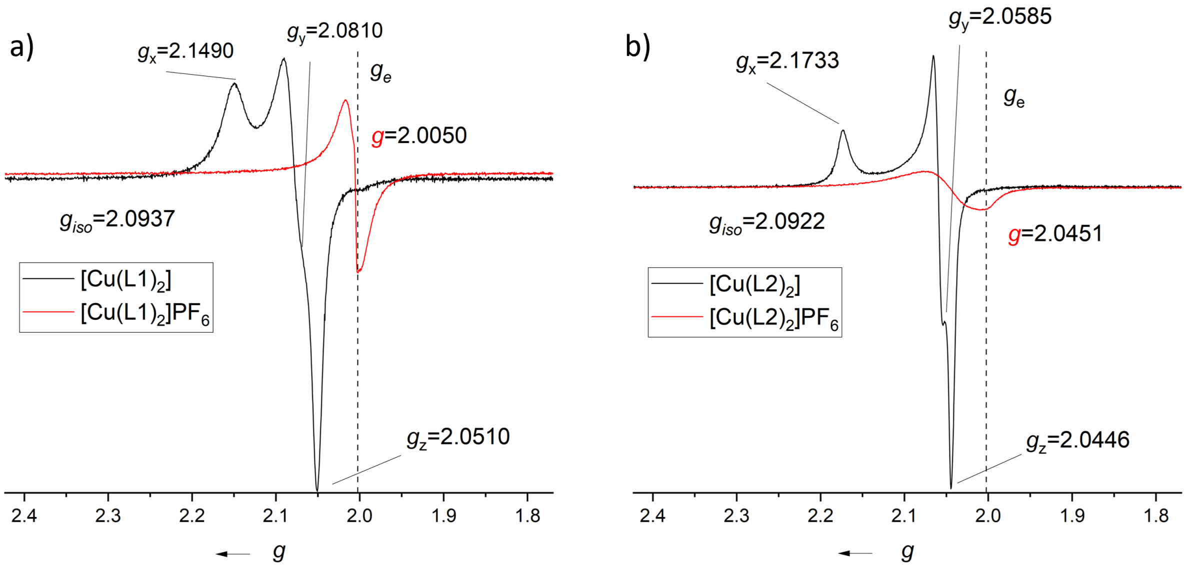

Figure S1. Comparison of the solid-state X-Band ESR spectra of **a)** $[Cu(L1)_2]$ (6.1 K) and $[Cu(L1)_2](PF_6)$ (6.2 K) and **b)**$[Cu(L2)_2]$ (5.8 K) and $[Cu(L2)_2](PF_6)$ (6.3 K).

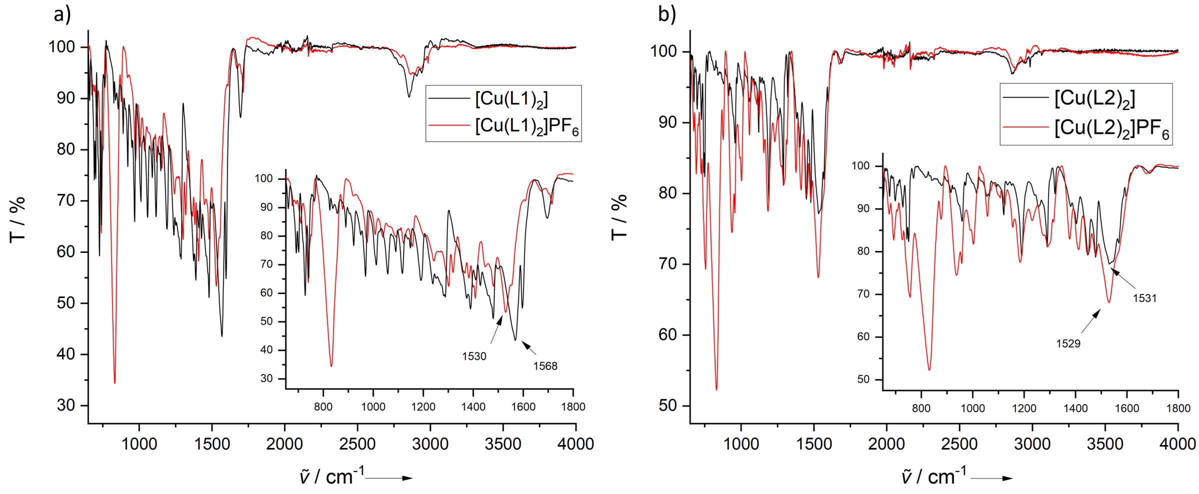

Figure S2. Comparison of the IR (ATR) spectra of **a)** $[Cu(L1)_2]$ and $[Cu(L1)_2](PF_6)$ and **b)** $[Cu(L2)_2]$ and $[Cu(L2)_2](PF_6)$.

3 Energy differences by Hartree-Fock and density functional calculations with different functionals and basis sets

In this section, a comparison between different density functionals using the two basis sets def2-SVP and def2-TZVP is made to evaluate if significant energy deviations for different states of $[Cu(L1)_2]^+$ and $[Cu(L2)_2]^+$ arise. The calculation were performed using ORCA[3] and TURBOMOLE.[4,5] The term ^3A denotes the triplet state, the term ^{BS}A the broken-symmetry open-shell term and the $^1A^{RHF/RKS}$ denotes the closed-shell state (Table S1). As can be observed, even though the different functionals reveal significant energy differences between the three states for a given basis set, no significant energy differences between the three states can be found when comparing def2-SVP and def2-TZVP. The methods HF and BHLYP reveal larger energy differences between the closed-shell $^1A^{RHF/RKS}$ and the triplet state ^3A compared to B3LYP. The inclusion of a dispersion correction (D3) changes the energetic difference only slightly in all scenarios.

Table S1. Relative energies of different electronic states of $[Cu(L1)_2]^+$ and $[Cu(L2)_2]^+$ from Hartree-Fock calculations and from density functional calculations with the BHLYP, B3LYP, CAM-B3LYP, TPSSh functionals and the def2-SVP and def2-TZVP basis sets without and with D3 dispersion correction and without or with inclusion of a dielectric environment. Additionally, calculations using the relativistic method ZORA were employed.

Complex	Method	ϵ_r	Sym.	Term	E_{el}^{SVP}/eV	E_{el}^{TZVP}/eV
$[Cu(L1)_2]^+$	HF		C_1	^3A	0	0
	HF		C_1	^{BS}A	0.0012	0.0010
	HF		C_1	$^1A^{RHF}$	2.5203	2.3133
	BHLYP		C_1	^3A	0	0
	BHLYP		C_1	^{BS}A	0.0014	0.0006
	BHLYP		C_1	$^1A^{RKS}$	1.3582	1.4178
	B3LYP		C_1	^3A	0	0
	B3LYP		C_1	^{BS}A	0.0092	0.0013
	B3LYP		C_1	$^1A^{RKS}$	0.5078	0.5654
	BHLYP+D3		C_1	^3A	0	0
	BHLYP+D3		C_1	^{BS}A	0.0032	0.0013
	BHLYP+D3		C_1	$^1A^{RKS}$	1.1166	1.1389
	B3LYP+D3		C_1	^3A	0	0
	B3LYP+D3		C_1	^{BS}A	0.0202	0.0116
	B3LYP+D3		C_1	$^1A^{RKS}$	0.3352	0.3211
	B3LYP+D3+COSMO	9.1	C_1	^3A	-	0
	B3LYP+D3+COSMO	9.1	C_1	^{BS}A	-	0.0143
	B3LYP+D3+COSMO	9.1	C_1	$^1A^{RKS}$	-	0.3031
	CAM-B3LYP+D3		C_1	^3A	-	0
	CAM-B3LYP+D3		C_1	^{BS}A	-	0.002
	CAM-B3LYP+D3		C_1	$^1A^{RKS}$	-	0.760
	TPSSh+D3		C_1	^3A	-	0
	TPSSh+D3		C_1	^{BS}A	-	0.037
	TPSSh+D3		C_1	$^1A^{RKS}$	-	0.197
	ZORA B3LYP+D3		C_1	^3A	-	0
	ZORA B3LYP+D3		C_1	^{BS}A	-	0.036
	ZORA B3LYP+D3		C_1	$^1A^{RKS}$	-	0.640
	ZORA TPSSh+D3		C_1	^3A	-	0
	ZORA TPSSh+D3		C_1	^{BS}A	-	0.041
	ZORA TPSSh+D3		C_1	$^1A^{RKS}$	-	-

Complex	Method	ϵ_r	Sym.	Term	E_{el}^{SVP}/eV	E_{el}^{TZVP}/eV
$[Cu(L2)_2]^+$	HF		C_1	^3A	0	0
	HF		C_1	^{BS}A	0.0067	0.0013
	HF		C_1	$^1A^{RHF}$	3.0589	2.4455
	BHLYP		C_1	^3A	0	0
	BHLYP		C_1	^{BS}A	0.0038	0.0026
	BHLYP		C_1	$^1A^{RKS}$	1.2532	1.2448
	B3LYP		C_1	^3A	0	0
	B3LYP		C_1	^{BS}A	0.0191	0.0117
	B3LYP		C_1	$^1A^{RKS}$	0.4432	0.4576
	BHLYP+D3		C_1	^3A	0	0
	BHLYP+D3		C_1	^{BS}A	0.0056	0.0045
	BHLYP+D3		C_1	$^1A^{RKS}$	0.9914	0.9830
	B3LYP+D3		C_1	^3A	0	0
	B3LYP+D3		C_1	^{BS}A	0.0269	0.0228
	B3LYP+D3		C_1	$^1A^{RKS}$	0.2724	0.2530
	B3LYP+D3+COSMO	9.1	C_1	^3A	-	0
	B3LYP+D3+COSMO	9.1	C_1	^{BS}A	-	0.0287
	B3LYP+D3+COSMO	9.1	C_1	$^1A^{RKS}$	-	0.2282
	CAM-B3LYP+D3		C_1	^3A	-	0
	CAM-B3LYP+D3		C_1	^{BS}A	-	0.012
	CAM-B3LYP+D3		C_1	$^1A^{RKS}$	-	0.607
	TPSSh+D3		C_1	^3A	-	0
	TPSSh+D3		C_1	^{BS}A	-	0.041
	TPSSh+D3		C_1	$^1A^{RKS}$	-	0.146
	ZORA B3LYP+D3		C_1	^3A	-	0
	ZORA B3LYP+D3		C_1	^{BS}A	-	0.054
	ZORA B3LYP+D3		C_1	$^1A^{RKS}$	-	0.580
	ZORA TPSSh+D3		C_1	^3A	-	0
	ZORA TPSSh+D3		C_1	^{BS}A	-	0.046
	ZORA TPSSh+D3		C_1	$^1A^{RKS}$	-	-

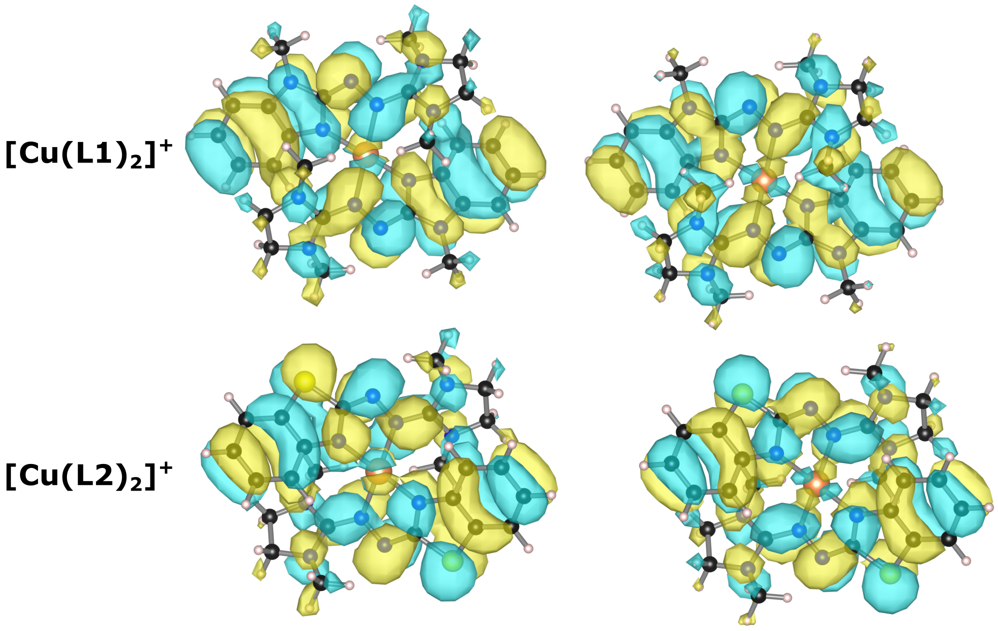

Figure S3. Singly occupied unrestricted natural orbitals for $[Cu(L1/L2)_2]^+$ in triplet state (B3LYP+D3/def2-TZVP).

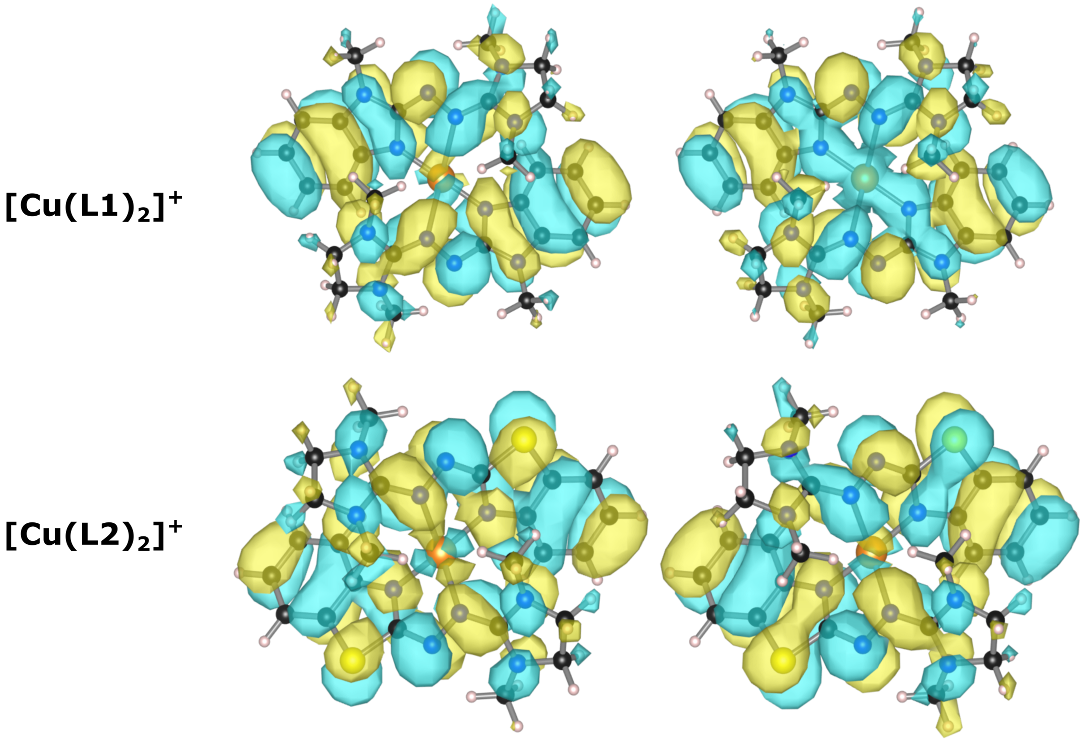

Figure S4. Illustration of highest singly occupied molecular orbitals (alpha) for both oxidized complexes using UKS CAM-B3LYP+D3 and def2-TZVP.

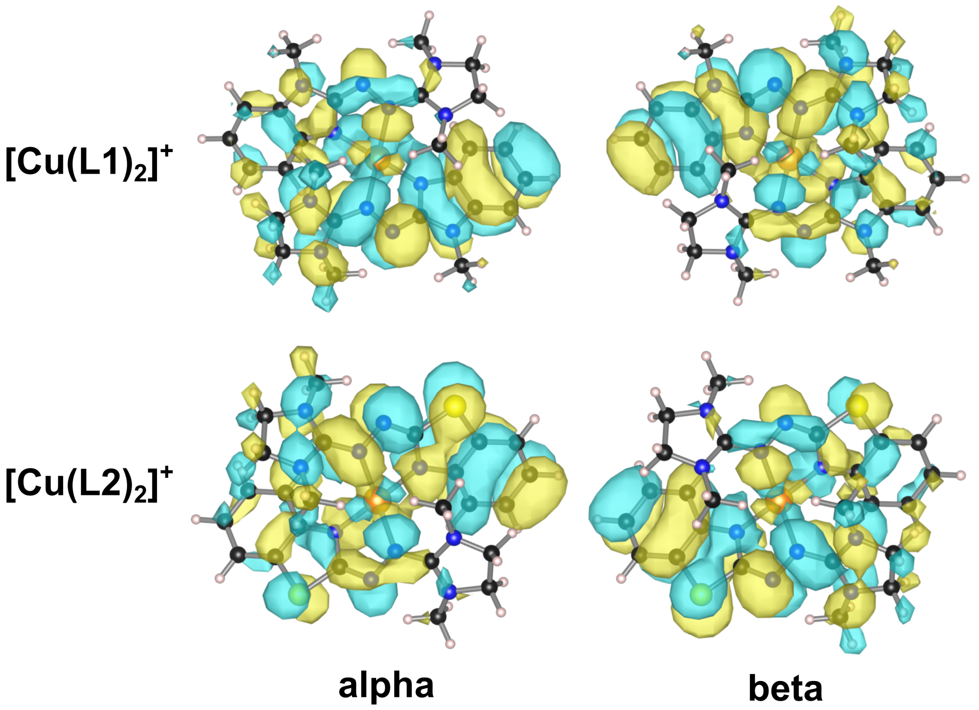

Figure S5. Illustration of highest singly occupied molecular orbitals for both oxidized complexes using broken-symmetry B3LYP+D3 and def2-TZVP.

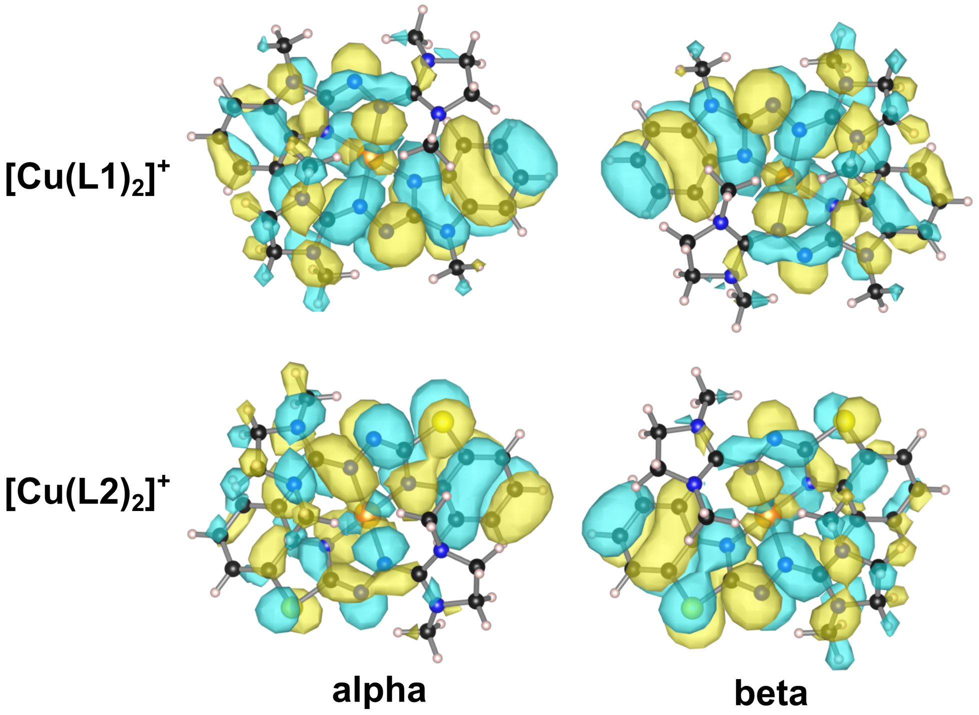

Figure S6. Illustration of highest singly occupied molecular orbitals for both oxidized complexes using broken-symmetry TPSSh+D3 and def2-TZVP.

Table S2. Expectation values $\langle \hat{S}^2 \rangle$ of the broken-symmetry (BS) state and the triplet state (high spin = HS) of the two complexes for different functionals.

Complex	Method	$\langle \hat{S}^2_{\mathrm{BS}} \rangle$	$\langle \hat{S}^2_{\mathrm{HS}} \rangle$
$[Cu(L1)_2]^+$	B3LYP	1.00	2.02
	CAM-B3LYP	1.03	2.03
	TPSSh	0.97	2.02
	ZORA B3LYP	0.98	2.02
	ZORA TPSSh	0.96	2.03
$[Cu(L2)_2]^+$	B3LYP	0.97	2.03
	CAM-B3LYP	1.04	2.05
	TPSSh	0.95	2.02
	ZORA B3LYP	0.95	2.03
	ZORA TPSSh	0.95	2.02

4 Bond parameters and spin populations by density functional calculations with and without dispersion correction

In this section the bond parameters (in Å) for the neutral and cationic complexes obtained by B3LYP calculations with and without the D3 dispersion correction are shown (Table S3). Additionally, the values of the spin population at the copper atom are given using the def2-SVP or def2-TZVP basis sets with and without the D3 correction (Table S4).

Even though the electronic properties do not change significantly, it is important to note that the copper−ligand bond distances change significantly when neglecting the dispersion correction. In particular, in the case of $[Cu(L1)_2]^+$, the Cu−N^3 distance (Figure S7) increases by 0.44 Å when discarding the D3 correction (2.61 Å vs. 2.17 Å, def2-TZVP). The stronger M−L bonds in neutral organometallic complexes when using a dispersion correction have been discussed in previous works.[6,7] Smaller differences in the metal−ligand bond distances between dispersion corrected and non-corrected values are also found for the neutral complexes.

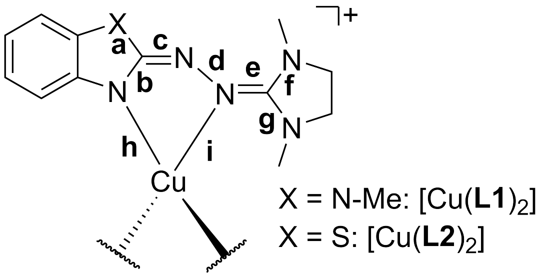

Figure S7. Ligand specific bond labels.

Table S3. Selected bond parameters (in Å) of the neutral and monocationic complexes by calculations with the B3LYP functional with the def2-SVP and def2-TZVP basis sets with and without D3 correction (all calculations are performed using the RIJCOSX appoximation and the def2/J auxillary basis set). Bond labels are given in Fig. S7.

$[Cu(L1)_2]$ Bond	def2-SVP	def2-TZVP	def2-SVP+D3	def2-TZVP+D3
a	1.3933	1.3883	1.3946	1.3897
b	1.3658	1.3632	1.3694	1.3672
c	1.3106	1.3063	1.3080	1.3028
d	1.3902	1.3985	1.3950	1.4045
e	1.3150	1.3091	1.3137	1.3087
f	1.3810	1.3795	1.3768	1.3763
g	1.3848	1.3831	1.3725	1.3362
h	1.9767	1.9554	1.9583	1.9405
i	2.1272	2.1419	2.0741	2.0757
$[Cu(L1)_2]^+$ Bond	def2-SVP	def2-TZVP	def2-SVP+D3	def2-TZVP+D3
a	1.3852	1.3790	1.3832	1.3782
b	1.3488	1.3453	1.3524	1.3485
c	1.3363	1.3400	1.3349	1.3327
d	1.3487	1.3388	1.3517	1.3532
e	1.3352	1.3302	1.3370	1.3325
f	1.3622	1.3629	1.3580	1.3565
g	1.3666	1.3627	1.3570	1.3513
h	1.9825	1.9223	1.9781	1.9557
i	2.2719	2.6158	2.1201	2.1735
$[Cu(L2)_2]$ Bond	def2-SVP	def2-TZVP	def2-SVP+D3	def2-TZVP+D3
a	1.7916	1.7782	1.7892	1.7771
b	1.3602	1.3559	1.3602	1.3607
c	1.2993	1.2973	1.3000	1.2952
d	1.3930	1.3989	1.3922	1.4020
e	1.3215	1.3128	1.3186	1.3132
f	1.3711	1.3750	1.3708	1.3704
g	1.3730	1.3797	1.3672	1.3616
h	1.9834	1.9620	1.9629	1.9477
i	2.0850	2.1168	2.0512	2.0523
$[Cu(L2)_2]^+$ Bond	def2-SVP	def2-TZVP	def2-SVP+D3	def2-TZVP+D3
a	1.7728	1.7573	1.7704	1.7566
b	1.3401	1.3351	1.3453	1.3409
c	1.3276	1.3306	1.3264	1.3263
d	1.3501	1.3460	1.3529	1.3533
e	1.3448	1.3395	1.3423	1.3396
f	1.3585	1.3569	1.3517	1.3499
g	1.3601	1.3596	1.3533	1.3469
h	2.0068	1.9747	1.9821	1.9684
i	2.1693	2.2814	2.0752	2.0908

Table S4. Mulliken spin populations of the copper atom using the B3LYP functional with the def2-SVP and def2-TZVP basis sets with and without D3 correction (all calculations are performed using the RIJCOSX approximation and the def2/J auxillary basis set). All calculations are performed for the triplet states.

Complex	def2-SVP	def2-TZVP	def2-SVP+D3	def2-TZVP+D3
$[Cu(L1)_2]$	0.40	0.41	0.43	0.46
$[Cu(L1)_2]^+$	0.11	0.03	0.20	0.16
$[Cu(L2)_2]$	0.43	0.41	0.44	0.45
$[Cu(L2)_2]^+$	0.17	0.09	0.25	0.22

Table S5. Mulliken spin populations of the copper atom using the different functional with the def2-TZVP basis set and D3 correction (all calculations are performed using the RIJCOSX approximation and the def2/J auxillary basis set). All calculations are performed for the triplet states.

Complex	B3LYP+D3	CAM-B3LYP+D3	TPSSh+D3	ZORA B3LYP+D3	ZORA TPSSh+D3
$[Cu(L1)_2]^+$	0.16	0.02	0.26	0.24	0.28
$[Cu(L2)_2]^+$	0.22	0.10	0.28	0.29	0.30

5 Absorption spectra by TDDFT calculations with the B3LYP functional

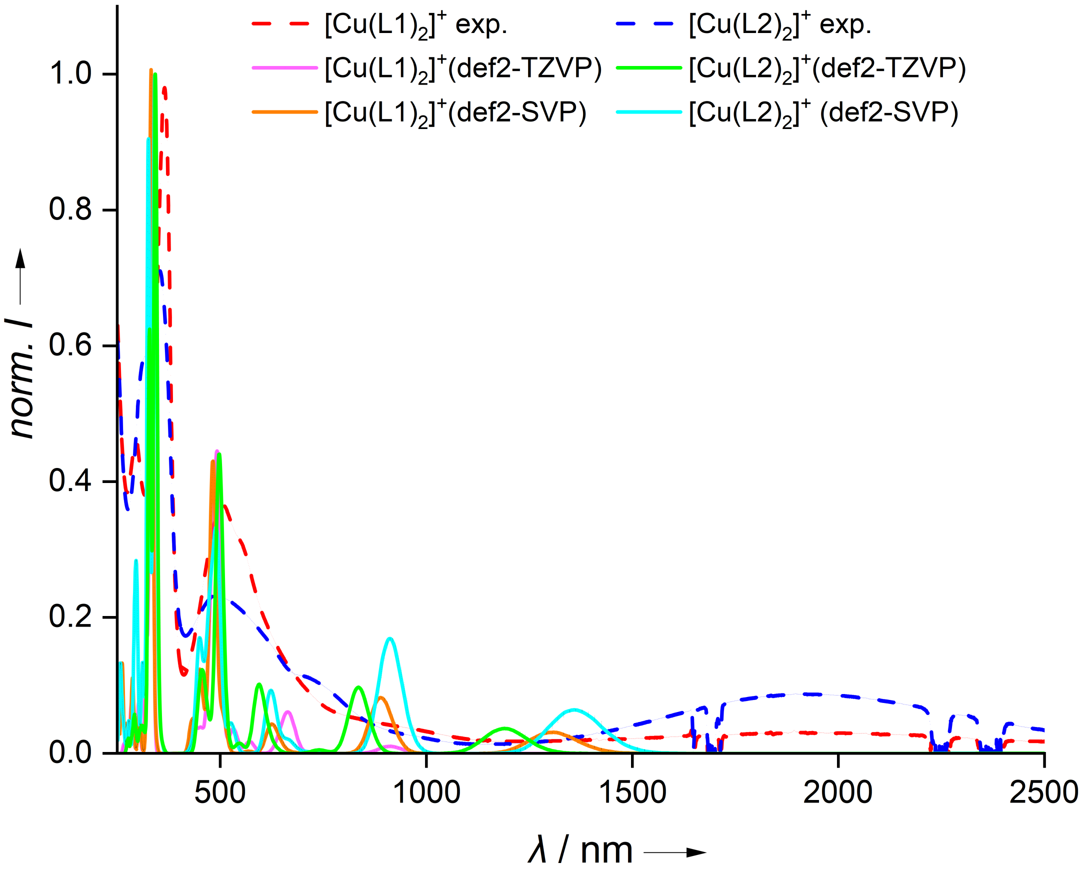

Figure S8. Electronic absorption spectra by TDDFT calculations (B3LYP/def2-TZVP and B3LYP/def2-SVP) for the triplet states of the $[Cu(L1)_2]^+$ and $[Cu(L2)_2]^+$ complexes obtained without inclusion of the D3 dispersion correction and experimental UV-vis spectra of the salts $[Cu(L1)_2]PF_6$ and $[Cu(L2)_2]PF_6$, measured in CH_2Cl_2.[1] The first 100 roots were calculated. The calculated transitions were fitted with gaussians of 0.1 eV width.

By TDDFT calculations at structures obtained without the D3 dispersion correction, spectra similar to the spectra for the structures obtained with dispersion correction are obtained (Figure S8), although the MLCT peak occurs at even shorter wavelengths. This discrepancy can be attributed to the shorter copper−ligand distances in the D3 corrected structures, resulting in an improved agreement with the experimental spectra. Hence, the inclusion of dispersion interactions for the monocationic mononuclear homoleptic copper complexes seems to be crucial and should not be overlooked.

6 Molecular orbitals of neutral $[Cu(L2)_2]$

Figure S9 illustrates the α and β MOs of the neutral $[Cu(L1)_2]$ complex, obtained by unrestricted B3LYP/def2-SVP calculations. There are two quasi-degenerate α MOs that are predominantly located at the ligands (MO 150 and 151). Furthermore, β MO 150 is the counterpart of α MO 151. However, the energy (-3.73 eV) is slightly higher compared to the highest α MOs (-3.98 eV / -3.99 eV). MOs α 149 and β 149 have larger copper contributions and differ significantly in energy (-5.46 eV and -4.23 eV) and shape. Inspecting β MO 149, it is found that the ligand part is similar to α 150 but there is also a clear copper contribution, absent in α 150. This mixing and the similar energy between the MOs 150 and α MO 151 may indicate a multiconfigurational character for the oxidized, monocationic species.[36] Figure S10 illustrates the molecular orbitals (MO) of $[Cu(L2)_2]$ by UKS B3LYP/def2-SVP calculations. Comparable to $[Cu(L1)_2]$, MO 149β is similar to 150α and MO 150β is similar to 151α. The mixed MOs indicate a possible multiconfigurational character when an electron is taken from the system.

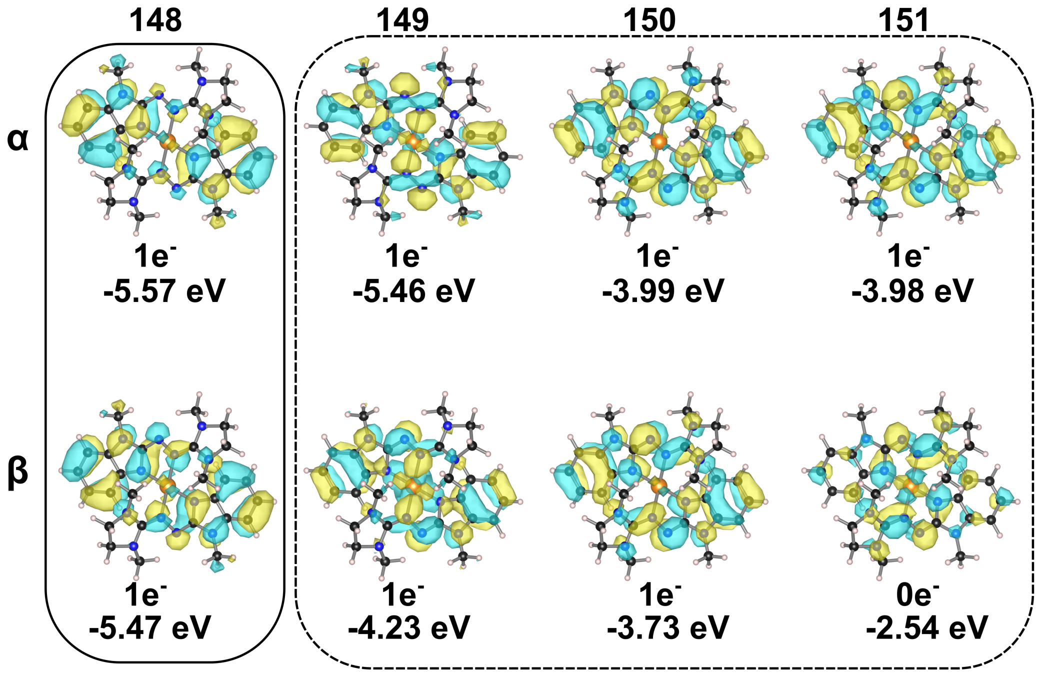

Figure S9. Illustration of the isodensity surfaces and energies of the molecular orbitals (α and β) of neutral $[Cu(L1)_2]$ by UKS-B3LYP/def2-SVP calculations. The dotted black frame shows the relevant mixed molecular orbitals.

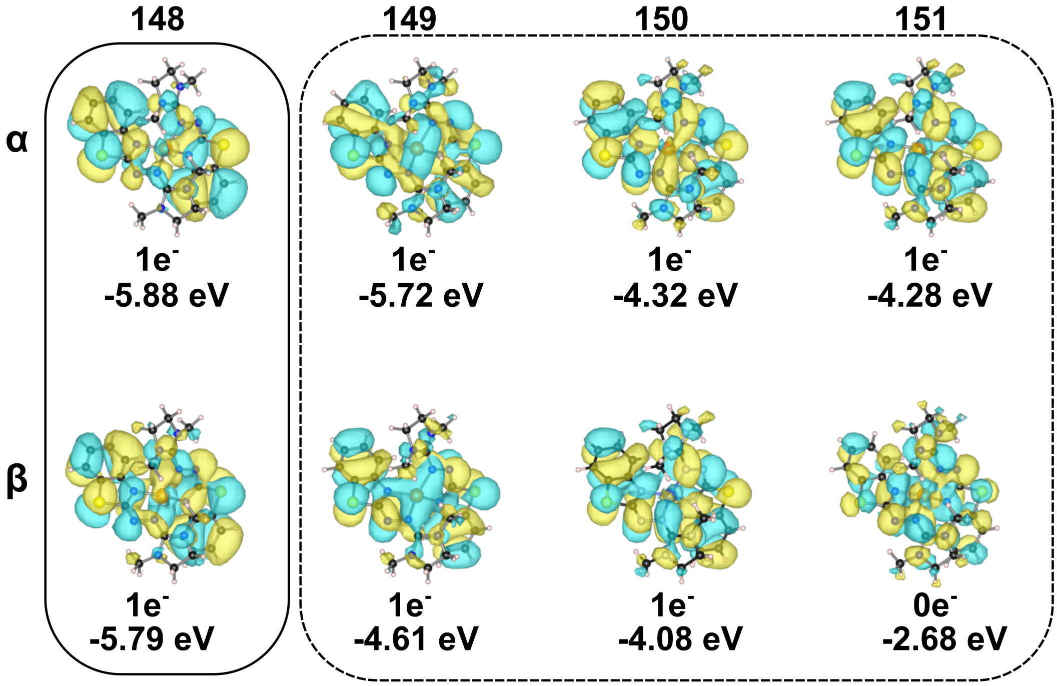

Figure S10. Illustration of the isodensity surfaces and energies of the molecular orbitals (α and β) of neutral $[Cu(L2)_2]$ by UKS B3LYP/def2-SVP calculations. The dotted black frame shows the relevant mixed molecular orbitals.

7 Bond parameters of the complexes $[Cu(L1)_2]^+$ and $[Cu(L2)_2]^+$ by CASSCF calculations

The bond distances of the optimized structures of $[Cu(L1)_2]^+$ and $[Cu(L2)_2]^+$ obtained by CASSCF(4,6) calculations are shown in Table S6, namely on the one hand the values for the structure optimized for the average of the [121] and [112] singlet states, denoted $([121]+[112])_{sym}$, and on the other hand the values for the structure of the [211] singlet state, denoted $[211]_{sym}$. The corresponding bonds are illustrated in Fig. S7. The bond i reveals a very large difference between the two structures, there are also significant differences for the bonds b, c, d, and h.

Table S6. Bond distances (Å) of the averaged structure of the [121] and [112] singlet states and of the structure of the [211] singlet state of the $[Cu(L1)_2]^+$ and $[Cu(L2)_2]^+$ complexes by CASSCF(4,6) calculations, and differences (Δ) between the two structures. Bond labels are given in Fig. S7.

Bond	$[Cu(L1)_2]^+$			$[Cu(L2)_2]^+$		
	$([121]+[112])_{sym}$	$[211]_{sym}$	Δ	$([121]+[112])_{sym}$	$[211]_{sym}$	Δ
a	1.3595	1.3527	-0.01	1.7455	1.7415	0.00
b	1.3330	1.3061	-0.03	1.3241	1.2852	-0.04
c	1.3228	1.3657	0.04	1.3170	1.3617	0.04
d	1.3659	1.3152	-0.05	1.3611	1.3167	-0.04
e	1.3204	1.3198	0.00	1.3281	1.3291	0.00
f	1.3416	1.3423	0.00	1.3376	1.3397	0.00
g	1.3433	1.3458	0.00	1.3420	1.3420	0.00
h	1.9930	2.0006	0.01	2.0051	2.0874	0.08
i	2.0903	2.7170	0.63	2.0757	2.4708	0.40

8 Leading configurations in the CASSCF(4,6) wavefunctions of the [121], [112], and [211] states of the $[Cu(L1)_2]^+$ and $[Cu(L2)_2]^+$ complexes

Tables S7 and S8 show the leading configurations and weights of the three calculated singlet roots of $[Cu(L1)_2]^+$ and $[Cu(L2)_2]^+$ obtained by SA-CASSCF(4,6) calculations at the averaged optimized structure (Weights of the roots: 0.25, 0.25, 0.5). ROOT 0 and ROOT 1 are approximately degenerate and belong to the [121] and [112] electronic singlet states while ROOT 2 corresponds to the [211] singlet state.

Table S7. Leading configurations and weights for the three calculated roots of $[Cu(L1)_2]^+$ by SA-CASSCF(4,6) calculations with the def2-SVP basis set. (weights: 0.25, 0.25, 0.5) The format is directly extracted from the ORCA output. The first column shows the weights of the configurations (in square brackets) and the last column shows the occupation of the molecular orbitals (between 0 and 2).

ROOT 0: E= -3295.0807578222 Eh
0.63604 [15]: 121000
0.15365 [45]: 022000
0.05706 [5]: 202000
0.03719 [46]: 021100
0.02551 [22]: 111001
0.02467 [16]: 120100
0.01649 [30]: 102010
0.01014 [0]: 220000
0.00845 [29]: 102100
0.00562 [4]: 210001
0.00409 [27]: 110011
0.00389 [49]: 020200
0.00284 [71]: 002200
ROOT 1: E= -3295.0802119023 Eh 0.015 eV 119.8 cm**-1
0.78470 [19]: 112000
0.07151 [55]: 012100
0.06444 [1]: 211000
0.03334 [21]: 111010
0.01319 [18]: 120001
0.00606 [3]: 210010
0.00508 [56]: 012010
0.00375 [36]: 101011
0.00346 [20]: 111100
0.00313 [26]: 110020
0.00303 [59]: 011110
ROOT 2: E= -3295.0570375025 Eh 0.645 eV 5206.0 cm**-1
0.46801 [0]: 220000
0.37622 [5]: 202000
0.03212 [29]: 102100
0.02377 [46]: 021100
0.02013 [16]: 120100
0.01650 [49]: 020200
0.01397 [71]: 002200
0.01120 [22]: 111001
0.01071 [45]: 022000
0.00922 [7]: 201010
0.00361 [4]: 210001
0.00311 [32]: 101200
0.00282 [15]: 121000

Table S8. Leading configurations and weights for the three calculated roots of $[Cu(L2)_2]^+$ by SA-CASSCF(4,6) calculations with the def2-SVP basis set. (weights: 0.25, 0.25, 0.5) The format is directly extracted from the ORCA output. The first column shows the weights of the configurations (in square brackets) and the last numbers show the occupation of the molecular orbitals (between 0 and 2).

ROOT 0: E= -3901.8648497487 Eh
0.81867 [19]: 112000
0.06972 [55]: 012100
0.03813 [22]: 111001
0.03162 [1]: 211000
0.01307 [17]: 120010
0.00555 [36]: 101011
0.00378 [28]: 110002
0.00299 [4]: 210001
0.00297 [60]: 011101
0.00293 [20]: 111100
ROOT 1: E= -3901.8642472035 Eh 0.016 eV 132.2 cm**-1
0.72153 [15]: 121000
0.09110 [45]: 022000
0.04771 [5]: 202000
0.04084 [46]: 021100
0.02856 [21]: 111010
0.01609 [16]: 120100
0.01532 [31]: 102001
0.00814 [29]: 102100
0.00556 [27]: 110011
0.00425 [3]: 210010
0.00289 [35]: 101020
0.00281 [49]: 020200
ROOT 2: E= -3901.8335406988 Eh 0.852 eV 6871.5 cm**-1
0.45252 [0]: 220000
0.40292 [5]: 202000
0.02853 [29]: 102100
0.02008 [16]: 120100
0.01580 [46]: 021100
0.01393 [49]: 020200
0.01299 [71]: 002200
0.01214 [15]: 121000
0.01150 [21]: 111010
0.00875 [8]: 201001
0.00660 [45]: 022000
0.00510 [3]: 210010

9 Validation of active space and basis set size

The following tables show the energy differences between the low-lying singlet and the triplet states with different basis sets (see Table S9) and different active spaces (see Table S10). The calculations rely on the B3LYP+D3/def2-TZVP structures. The change of basis set size leads to only small differences in the relative energies. For the study of different active spaces, for convenience the def2-SVP basis set is used. When increasing the size of the active space, the relative energies change only slightly. For instance, for the [121] ^{3}A and [121] ^{1}A excited states of the $[Cu(L1)_2]^+$ complex, the NEVPT2 calculations with the CAS(4,6) active space yield relative energies with respect to the [112] ^{1}A ground state of 0.0078 and 0.207 eV, whereas with the CAS(12,14) space values of 0.015 and 0.289 eV, respectively, are obtained. Thus, the CAS(4,6) space indeed is suitable for further investigations. Additionally, Table S11 shows the calculated energy differences of all configurations considering relativistic effects using ZORA/ZORA-TZVP. The energy differences slightly change, however, similar trends as for the non-relativistic case can be found.

Table S9. Total energies and relative energies of the low-lying singlet and triplet states of the $[Cu(L1)_1]^+$ and $[Cu(L2)_2]^+$ complexes by CASSCF and NEVPT2 calculations at the B3LYP+D3/def2-TZVP structures for different basis sets. For the three lowest-lying states of each multiplicity, weights of 0.25, 0.25, and 0.5 are chosen.

Complex	Basis	Mult.	Conf.	CAS(4,6)		SC-NEVPT2	
				Energy / a.u.	Δ E / eV	Energy / a.u.	Δ E / eV
$[Cu(L1)_2]^+$	Def2-SVP	1	[121]	-3295.0736342	0.00	-3300.857768	0.00
			[112]	-3295.0779255	-0.12	-3300.865374	-0.21
			[211]	-3295.0236555	1.36	-3300.856459	0.04
		3	[121]	-3295.0780380	-0.12	-3300.865110	-0.20
			[112]	-3295.0721392	0.04	-3300.855033	0.07
			[211]	-3295.0236401	1.36	-3300.856069	0.05
	Def2-TZVP	1	[121]	-3297.0943591	0.00	-3304.262658	0.00
			[112]	-3297.0987414	-0.12	-3304.271265	-0.23
			[211]	-3297.0430098	1.40	-3304.267421	-0.13
		3	[121]	-3297.0982990	-0.11	-3304.270092	-0.20
			[112]	-3297.0933867	0.03	-3304.260603	0.06
			[211]	-3297.0429798	1.40	-3304.266891	-0.12
	Def2-TZVPP	1	[121]	-3297.1096065	0.00	-3304.525210	0.00
			[112]	-3297.1138714	-0.12	-3304.533951	-0.24
			[211]	-3297.0566852	1.44	-3304.533518	-0.23
		3	[121]	-3297.1133887	-0.10	-3304.532722	-0.20
			[112]	-3297.1086687	0.03	-3304.523159	0.06
			[211]	-3297.0566646	1.44	-3304.532995	-0.21
	Def2-QZVPP	1	[121]	-3297.220447	0.00	-3305.097915	0.00
			[112]	-3297.224630	-0.11	-3305.106207	-0.23
			[211]	-3297.165672	1.49	-3305.101927	-0.11
		3	[121]	-3297.224017	-0.10	-3305.104783	-0.19
			[112]	-3297.219636	0.02	-3305.096025	0.05
			[211]	-3297.165653	1.49	-3305.101421	-0.10

Complex	Basis	Mult.	Conf.	CAS(4,6)		SC-NEVPT2	
				Energy / a.u.	Δ E / eV	Energy / a.u.	Δ E / eV
$[Cu(L2)_2]^+$	Def2-SVP	1	[121]	-3901.857390	0.00	-3907.302149	0.00
			[112]	-3901.861938	-0.12	-3907.310229	-0.22
			[211]	-3901.789904	1.84	-3907.291678	0.28
		3	[121]	-3901.862550	-0.14	-3907.310157	-0.22
			[112]	-3901.854273	0.08	-3907.296898	0.14
			[211]	-3901.789784	1.84	-3907.290835	0.31
	Def2-TZVP	1	[121]	-3903.961876	0.00	-3910.991680	0.00
			[112]	-3903.966430	-0.12	-3911.001148	-0.26
			[211]	-3903.892226	1.90	-3910.991636	0.00
		3	[121]	-3903.966455	-0.12	-3910.999821	-0.22
			[112]	-3903.959349	0.07	-3910.987302	0.12
			[211]	-3903.892158	1.90	-3910.990741	0.03
	Def2-TZVPP	1	[121]	-3903.979781	0.00	-3910.740929	0.00
			[112]	-3903.975360	0.12	-3910.750271	-0.25
			[211]	-3903.903805	2.07	-3910.737437	0.10
		3	[121]	-3903.979756	0.00	-3910.749026	-0.22
			[112]	-3903.972882	0.19	-3910.736543	0.12
			[211]	-3903.903758	2.07	-3910.736531	0.12
	Def2-QZVPP	1	[121]	-3904.099835	0.00	-3911.559825	0.00
			[112]	-3904.104158	-0.12	-3911.568938	-0.25
			[211]	-3904.026112	2.01	-3911.555402	0.12
		3	[121]	-3904.103994	-0.11	-3911.567404	-0.21
			[112]	-3904.097505	0.06	-3911.555693	0.11
			[211]	-3904.026080	2.01	-3911.554545	0.14

Table S10. Total energies and relative energies of the low-lying singlet and triplet states of the $[Cu(L1)_1]^+$ and $[Cu(L2)_2]^+$ complexes by CASSCF and NEVPT2 calculations at the B3LYP+D3/def2-TZVP structures (def2-SVP basis set). For the three lowest-lying states of each multiplicity, weights of 0.25, 0.25, and 0.5 are chosen.

Complex	Space	Mult.	Conf.	CAS(X,X) Energy / a.u.	ΔE / eV	SC-NEVPT2 Energy / a.u.	ΔE / eV
$[Cu(L1)_2]^+$	4,6	1	[121]	-3295.073634	0.00	-3300.857768	0.00
			[112]	-3295.077925	-0.12	-3300.865374	-0.21
			[211]	-3295.023655	1.36	-3300.856459	0.04
		3	[121]	-3295.078038	-0.12	-3300.865110	-0.20
			[112]	-3295.072139	0.04	-3300.855033	0.07
			[211]	-3295.023640	1.36	-3300.856068	0.05
	8,8	1	[121]	-3295.097895	0.00	-3300.844963	0.00
			[112]	-3295.104525	-0.18	-3300.856558	-0.32
			[211]	-3295.054839	1.17	-3300.846392	-0.04
		3	[121]	-3295.104455	-0.18	-3300.855900	-0.30
			[112]	-3295.096568	0.04	-3300.841896	0.08
			[211]	-3295.054743	1.17	-3300.845558	-0.02
	8,10	1	[121]	-3295.113002	0.00	-3300.819311	0.00
			[112]	-3295.117861	-0.13	-3300.829438	-0.28
			[211]	-3295.069470	1.18	-3300.818812	0.01
		3	[121]	-3295.119495	-0.18	-3300.829451	-0.28
			[112]	-3295.110221	0.08	-3300.815918	0.09
			[211]	-3295.069679	1.18	-3300.818635	0.02
	10,10	1	[121]	-3295.115654	0.00	-3300.830126	0.00
			[112]	-3295.122171	-0.18	-3300.841363	-0.31
			[211]	-3295.072684	1.17	-3300.830853	-0.02
		3	[121]	-3295.122269	-0.18	-3300.840408	-0.28
			[112]	-3295.114053	0.04	-3300.827599	0.07
			[211]	-3295.072621	1.17	-3300.830026	0.00
	12,12	1	[121]	-3295.133730	0.00	-3300.817800	0.00
			[112]	-3295.139523	-0.16	-3300.828955	-0.30
			[211]	-3295.090602	1.17	-3300.816908	0.02
		3	[121]	-3295.139876	-0.17	-3300.827359	-0.26
			[112]	-3295.131769	0.05	-3300.815628	0.06
			[211]	-3295.090566	1.17	-3300.816064	0.05
	12,14	1	[121]	-3295.150406	0.00	-3300.820206	0.00
			[112]	-3295.155654	-0.14	-3300.830828	-0.29
			[211]	-3295.107600	1.16	-3300.819136	0.03
		3	[121]	-3295.156949	-0.18	-3300.830260	-0.27
			[112]	-3295.147765	0.07	-3300.817363	0.08
			[211]	-3295.107743	1.16	-3300.818765	0.04

Complex	Space	Mult.	Conf.	CAS(X,X)		SC-NEVPT2	
				Energy / a.u.	ΔE / eV	Energy /a.u.	ΔE / eV
$[Cu(L2)_2]^+$	4,6	1	[121]	-3901.857390	0.00	-3907.302149	0.00
			[112]	-3901.861938	-0.12	-3907.310229	-0.22
			[211]	-3901.789904	1.84	-3907.291678	0.28
		3	[121]	-3901.862550	-0.14	-3907.310157	-0.22
			[112]	-3901.854273	0.08	-3907.296898	0.14
			[211]	-3901.789785	1.84	-3907.290835	0.31
	8,8	1	[121]	-3901.886009	0.00	-3907.290190	0.00
			[112]	-3901.893627	-0.21	-3907.301505	-0.31
			[211]	-3901.830462	1.51	-3907.278384	0.32
		3	[121]	-3901.894257	-0.22	-3907.301354	-0.30
			[112]	-3901.883035	0.08	-3907.284388	0.16
			[211]	-3901.830231	1.52	-3907.277017	0.36
	8,10	1	[121]	-3901.887114	0.00	-3907.295193	0.00
			[112]	-3901.820645	1.81	-3907.303513	-0.23
			[211]	-3901.820756	1.81	-3907.285714	0.26
		3	[121]	-3901.892342	-0.14	-3907.303400	-0.22
			[112]	-3901.884052	0.08	-3907.290014	0.14
			[211]	-3901.820645	1.81	-3907.284890	0.28
	10,10	1	[121]	-3901.902068	0.00	-3907.298030	0.00
			[112]	-3901.909619	-0.21	-3907.286795	0.31
			[211]	-3901.845847	1.53	-3907.275629	0.61
		3	[121]	-3901.910141	-0.22	-3907.297834	0.01
			[112]	-3901.899141	0.08	-3907.281138	0.46
			[211]	-3901.845611	1.54	-3907.274281	0.65
	12,12	1	[121]	-3901.918401	0.00	-3907.284309	0.00
			[112]	-3901.925718	-0.20	-3907.295358	-0.30
			[211]	-3901.861083	1.56	-3907.273278	0.30
		3	[121]	-3901.926137	-0.21	-3907.295084	-0.29
			[112]	-3901.915491	0.08	-3907.278698	0.15
			[211]	-3901.860841	1.57	-3907.271919	0.34
	12,14	1	[121]	-3901.938616	0.00	-3907.285583	0.00
			[112]	-3901.944594	-0.16	-3907.295977	-0.28
			[211]	-3901.874837	1.74	-3907.277033	0.23
		3	[121]	-3901.945338	-0.18	-3907.295351	-0.27
			[112]	-3901.935404	0.09	-3907.280353	0.14
			[211]	-3901.874707	1.74	-3907.275906	0.26

Table S11. Total energies and relative energies of the low-lying singlet and triplet states of the $[Cu(L1)_1]^+$ and $[Cu(L2)_2]^+$ complexes by ZORA CASSCF (CAS(4,6)) calculations at the B3LYP+D3/def2-TZVP structures (ZORA-TZVP basis set). For the three lowest-lying states of each multiplicity, weights of 0.25, 0.25, and 0.5 are chosen.

Complex	Mult.	Conf.	Energy / a.u.	ΔE / eV
$[Cu(L1)_2]^+$	1	[121]	-3320.628858	0.00
		[112]	-3320.632949	-0.11
		[211]	-3320.559233	1.89
	3	[121]	-3320.632498	-0.10
		[112]	-3320.627287	0.04
		[211]	-3320.559221	1.89
$[Cu(L2)_2]^+$	1	[121]	-3930.809531	0.00
		[112]	-3930.813464	-0.11
		[211]	-3930.723997	2.33
	3	[121]	-3930.813809	-0.12
		[112]	-3930.806148	0.09
		[211]	-3930.723942	2.33

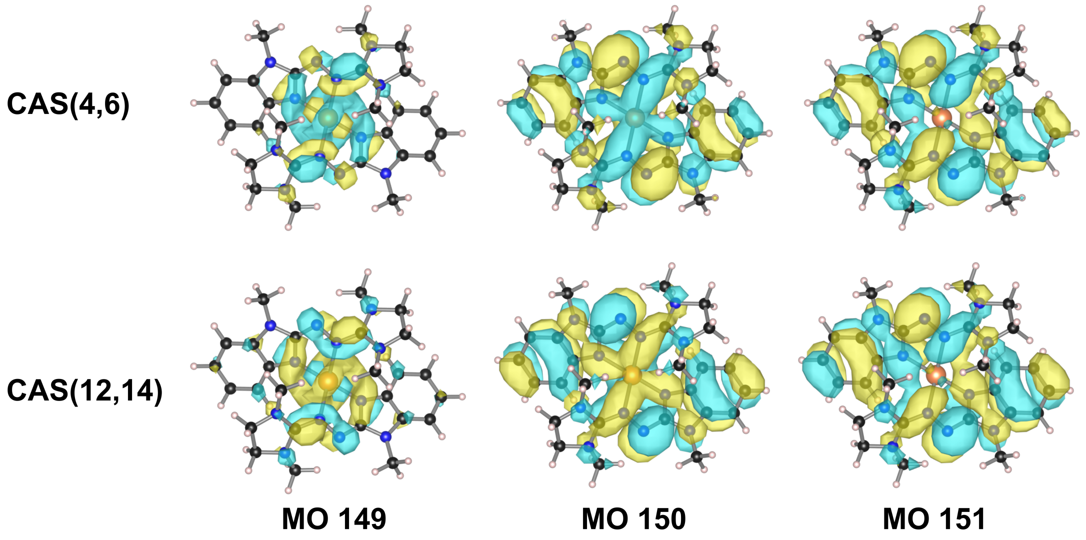

Figure S11. Comparison of the three occupied orbitals for $[Cu(L1)_2]^+$ using a CAS(4,6) and a CAS(12,14) space.

10 Structure optimization with CASSCF and inclusion of dynamic correlation

In this section a discussion of the CASSCF structure optimization is provided. The structures were optimized, on the one hand, for the [211] ^{1}A state (rather corresponding to Cu(I), $\mathbf{B}^+$), and on the other hand, for the average of the [121] and [112] ^{1}A states (both rather corresponding to Cu(II), $\mathbf{A}^+_{\mathrm{sym}}$). The [121] and [112] states have been treated together to avoid symmetry breaking of the wave function. Salient bond lengths and the energy differences for the optimized CASSCF structures of both complexes are collected in Table S12 and S13. For both complexes, the [211] state reveals considerably larger bond lengths between N^3 (the N atom of the central N_2 unit bound to Cu, see Table 1) and the copper atom (N^3−Cu), namely values of 2.717 Å (for $[Cu(L1)_2]^+$) and 2.471 Å (for $[Cu(L2)_2]^+$) compared with the values of 2.090 Å (for $[Cu(L1)_2]^+$) and 2.076 Å (for $[Cu(L2)_2]^+$) obtained for the average of the [121] and [112] states ($\mathbf{A}^+_{\mathrm{sym}}$). Besides that, large deviations are also observed when comparing the calculated results with the values of the crystal structure (2.095 Å for $[Cu(L1)_2]^+$ and 1.986/1.962 Å for $[Cu(L2)_2]^+$). The other bond parameters of the [211] state differ less from the values of the average structure of the [121] and [112] states and also from the crystal structure. For both cationic complexes, the bond parameters obtained for the averaged ([121]+[112]) structure are closer to the experimental crystal structure. Despite the large structural differences, according to the CASSCF calculations the three states of the $[Cu(L1)_2]^+$ complex are very close in energy. The [121] and [112] ^{1}A states have energies of −0.01 and 0.01 eV with respect to the [211] ^{1}A state, whereas in the case of the $[Cu(L2)_2]^+$ complex, the [121] and [112] ^{1}A states are found 0.17 and 0.17 eV below the [211] ^{1}A state (see Table S13).

To account for the dynamical electron correlation, single-point calculations using three different multireference methods (DCD-CAS(2), NEVPT2 and CASPT2) were carried out at the CASSCF(4,6) optimized structures. By inclusion of dynamical correlation, significant energy changes are found. The [121] and [112] ^{1}A states are strongly disfavored, with relative energies between 0.47 and 1.36 eV with respect to the [211] ^{1}A state (Table

Table S12. Bond parameters of the different electronic ^{1}A states by CASSCF(4,6)/def2-SVP calculations, compared to the experimental crystal structure values for $[Cu(L1)_2]^+$ and $[Cu(L2)_2]^+$ (see Figure 3 for notation). A more detailed structural comparison is included in the Supporting Information, FigureS6.

Complex	Structure	N^3-Cu / Å	N^1-Cu / Å	N^2-N^3 / Å	N^1-C^1 / Å
	[211]	2.717	2.001	1.315	1.306
$[Cu(L1)_2]^+$	([121]+[112])	2.090	1.993	1.366	1.333
	$Crystal_{exp}$	2.095(3)	1.933(3)	1.369(4)	1.343(5)
	[211]	2.4708	2.087	1.317	1.285
$[Cu(L2)_2]^+$	([121]+[112])	2.076	2.005	1.361	1.324
	$Crystal_{exp}$	1.986(3) / 1.962(3)	1.909(3) / 1.922(3)	1.405(4) / 1.371(4)	1.367(4) / 1.351(4)

Table S13. Relative energies of the [211], [121], and [112] ^{1}A states of $[Cu(L1)_2]^+$ and $[Cu(L2)_2]^+$ using different multireference methods at corresponding CASSCF structures (def2-SVP). Energies are given in eV.

Complex	Structure	State	CAS(4,6)	DCD-CAS(2)	NEVPT2	CASPT2
$[Cu(L1)_2]^+$	[211]	[211]	0.00	0.00	0.00	0.00
	([121]+[112])	[121]	−0.01	1.03	0.94	0.64
	([121]+[112])	[112]	0.01	1.36	0.92	0.71
$[Cu(L2)_2]^+$	[211]	[211]	0.00	0.00	0.00	0.00
	([121]+[112])	[121]	−0.17	0.71	0.78	0.47
	([121]+[112])	[112]	−0.17	1.03	0.73	0.53

S13). For both complexes, CASPT2 yields the smallest relative energies whereas NEVPT2 shows significantly higher values. The deviation between both methods was already discussed in previous works.[8] However, the surprisingly large changes in relative energies due to the inclusion of dynamic correlation indicate that very likely (and not unexpectedly) the CASSCF(4,6) structures are not close to the structures that would be obtained by methods including dynamical correlation. Thus, it is also desirable to determine the structures by multireference correlation methods to reliably confirm the relative energies or approach the correct values.

11 2D surfaces by CASSCF(4,6)/def2-SVP calculations

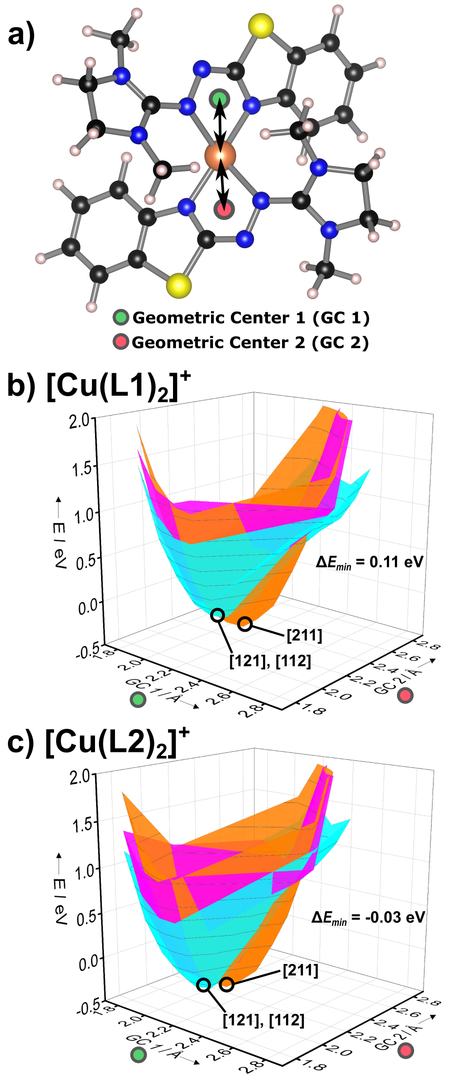

Figure S12. a) Illustration of the trajectories used for the PES scans, i. e. the shifts of the centroids of each ligand with respect to the Cu atom. The centroids for each ligand are defined by the four ligand atoms that are close to the copper atom, respectively. b) and c) PES plots for $[Cu(L1)_2]^+$ and $[Cu(L2)_2]^+$ from NEVPT2/CASSCF(4,6) calculations (41 scan points for each surface). The corresponding geometric center (GC) in a) are assigned with green and red dots. The [121] and [112] surfaces are shown in cyan and magenta, respectively, the [211] surface is shown in orange. ΔE_{min} indicates the energy difference between the minimum of the lower-lying one of the [121] and [112] states and the minimum of the [211] state (positive values mean that [211] is more stable). The weights of the three roots of singlet multiplicity were set to 0.25, 0.25 and 0.50, respectively.

12 Calculated structures of further $[Cu(X)_2]^+$ complexes

Figure S13 illustrates the structures used for the determination of the relative energies of the [121], [112], and [211] electronic states of the complexes of Cu with the modified ligands. The structures were obtained by ...

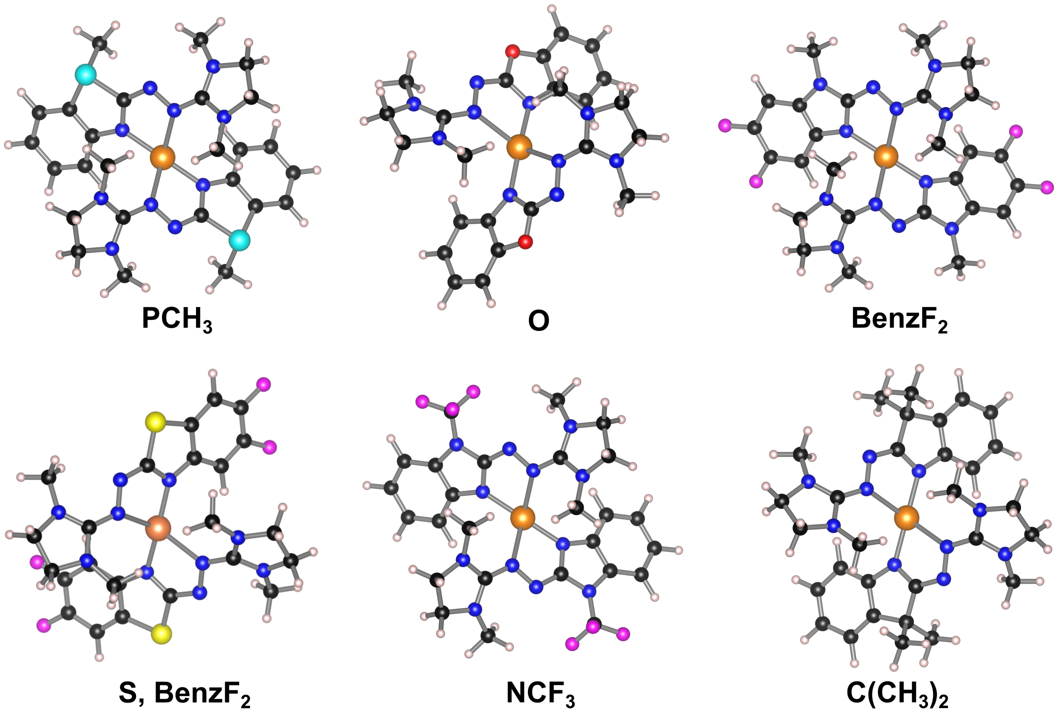

Figure S13. Structures of the complexes of Cu with the modified ligands used in the determination of energy differences between the [121], [112], and [211] states.

13 Calculated energies of $[Cu(X)_2]^+$ complexes with further ligands

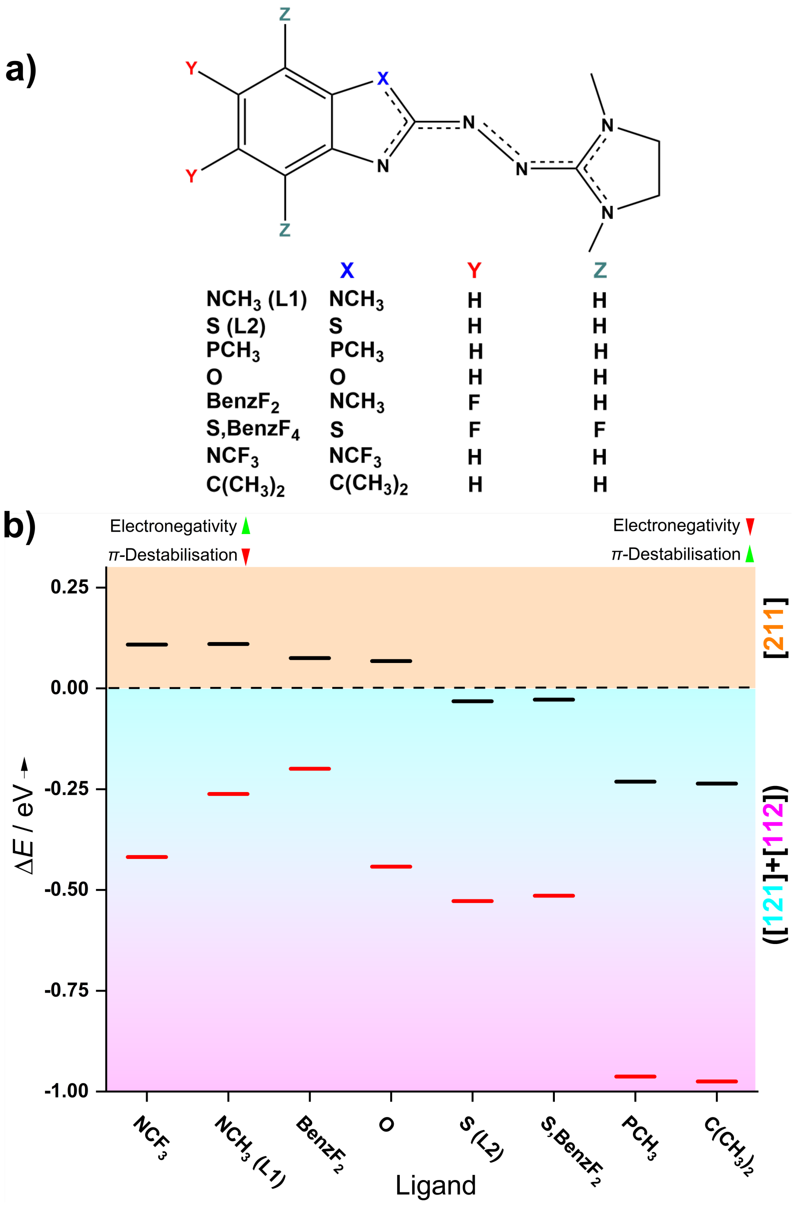

Figure S14. **a)** Schematic representation of the functional groups of different ligands. Details about the structures are found in Figure S13. **b)** Energy differences between the lower-lying one of the [121] and [112] singlet states, and the [211] singlet state for the different copper complexes (positive values means [211] is more stable). The functional groups are sorted into two groups according to their electronegativity and π-destabilisation. Green arrows indicate a strong effect while red arrows indicate a weak effect of the respective functional group. The black lines show the energy difference extracted from NEVPT2-CASSCF(4,6)/def2-SVP PES scans similar to Fig. S12, while the red lines illustrate the energy difference between the states at the corresponding B3LYP+D3 structure with NEVPT2-CASSCF(4,6)/def2-SVP.

14 Energies of the cationic complexes by multireference calculations

Table S14. Relative energies of the [211], [121], and [112] singlet states of $[Cu(L1)_2]^+$ and $[Cu(L2)_2]^+$ at the corresponding triplet B3LYP+D3/def2-TZVP structures by different multireference methods. Energies are given in eV.

Complex	State	CAS(4,6)	NEVPT2	DCD-CAS(2)	CASPT2
$[Cu(L1)_2]^+$	[121]	0.00	0.00	0.00	0.00
	[112]	0.13	0.22	0.56	0.08
	[211]	1.49	0.26	0.62	0.41
$[Cu(L2)_2]^+$	[121]	0.00	0.00	0.00	0.00
	[112]	0.14	0.24	0.53	0.21
	[211]	1.97	0.53	0.87	0.81

15 Total energies of different states of $[Cu(L1)_2]^+$ and $[Cu(L2)_2]^+$ by CASSCF and NEVPT2 calculations

Table S15. Total energies of the different low-lying states of $[Cu(L1)_2]^+$ and $[Cu(L2)_2]^+$ by CASSCF and NEVPT2 calculations with the def2-SVP basis set at different structures. The state averaged CASSCF calculations averaged the three lowest-lying singlet and triplet states. The weights for the three lowest-lying states of each multiplicity were 0.25, 0.25, and 0.5.

Complex	Structure	State	$E^{CASSCF(4,6)}$/Hartree		E^{NEVPT2}/Hartree	
			singlet	triplet	singlet	triplet
$[Cu(L1)_2]^+$	SA-CAS(4,6)	[121]	-3295.08116	-3295.08397	-3300.82796	-3300.82983
	SA-CAS(4,6)	[112]	-3295.08084	-3295.07802	-3300.82298	-3300.82045
	SA-CAS(4,6)	[211]	-3295.05814	-3295.05862	-3300.83767	-3300.83916
	B3LYP+D3	[121]	-3295.07785	-3295.07796	-3300.86527	-3300.86499
	B3LYP+D3	[112]	-3295.07355	-3295.07208	-3300.85765	-3300.85493
	B3LYP+D3	[211]	-3295.02372	-3295.02370	-3300.85636	-3300.85597
	$Crystal_{exp}$	[121]	-3294.71229	-3294.71268	-3300.44108	-3300.44181
	$Crystal_{exp}$	[112]	-3294.70826	-3294.70596	-3300.43475	-3300.43003
	$Crystal_{exp}$	[211]	-3294.64533	-3294.64518	-3300.43160	-3300.43089
$[Cu(L2)_2]^+$	SA-CAS(4,6)	[121]	-3901.86518	-3901.86854	-3907.26340	-3907.26987
	SA-CAS(4,6)	[112]	-3901.86518	-3901.86108	-3907.26686	-3907.25936
	SA-CAS(4,6)	[211]	-3901.83442	-3901.83495	-3907.27505	-3907.27644
	B3LYP+D3	[121]	-3901.86186	-3901.86244	-3907.31003	-3907.30992
	B3LYP+D3	[112]	-3901.86186	-3901.85421	-3907.30188	-3907.29672
	B3LYP+D3	[211]	-3901.78998	-3901.78987	-3907.29159	-3907.29075
	$Crystal_{exp}$	[121]	-3901.54427	-3901.54028	-3906.95684	-3906.95010
	$Crystal_{exp}$	[112]	-3901.52545	-3901.52269	-3906.93975	-3906.93408
	$Crystal_{exp}$	[211]	-3901.43181	-3901.43128	-3906.91004	-3906.90774

Table S16. Total energies of different low-lying states of $[Cu(L1)_2]^+$ and $[Cu(L2)_2]^+$ by CASSCF and NEVPT2 calculations with the def2-.VP basis set at different structures, namely on the one hand at the CASSCF structures of the [211] state singlet and on the other hand at the averaged structure of the [121] and [112] singlet states, and furthermore at the crystal structure. The state averaged CASSCF calculations averaged the three lowest-lying singlet and triplet states. The weights for the three lowest-lying states of each multiplicity were 0.25, 0.25, and 0.5.

Complex	Structure	State	$E^{CASSCF(4,6)}$/Hartree		E^{NEVPT2}/Hartree	
			singlet	triplet	singlet	triplet
$[Cu(L1)_2]^+$	[211]	[211]	-3295.11009	-3295.11010	-3300.87172	-3300.87160
	([121]+[112])	[121]	-3295.11048	-3295.11230	-3300.83700	-3300.83730
	([121]+[112])	[112]	-3295.10985	-3295.10669	-3300.83792	-3300.82997
	$Crystal_{exp}$	[121]	-3294.72350	-3294.72345	-3300.43140	-3300.43091
$[Cu(L2)_2]^+$	[211]	[211]	-3901.88797	-3901.88798	-3907.30063	-3907.30038
	([121]+[112])	[121]	-3901.89438	-3901.89669	-3907.27191	-3907.27080
	([121]+[112])	[112]	-3901.89420	-3901.89001	-3907.27393	-3907.26192
	$Crystal_{exp}$	[121]	-3901.55484	-3901.55006	-3906.94751	-3906.93895

16 Coordinates of [Cu(L1)$_2$] and [Cu(L2)$_2$]

In the following, the Cartesian coordinates of the neutral species and triplet states of the cations of [Cu(L1)$_2$] and [Cu(L2)$_2$] by B3LYP/def2-SVP calculations are shown.

Table S17. Coordinates of neutral [Cu(L1)$_2$] by B3LYP/def2-SVP calculations.

Atom	x [Å]	y [Å]	z [Å]
Cu	0.000	0.000	0.000
N	-1.946	0.749	-0.423
N	-1.640	1.765	-2.528
N	-3.697	0.954	-2.106
N	-2.869	0.349	0.537
N	-0.953	-0.564	1.637
N	-2.917	-0.502	2.759
C	-2.422	1.105	-1.595
C	-2.479	2.264	-3.607
H	-1.973	2.191	-4.583
H	-2.740	3.330	-3.436
C	-3.712	1.370	-3.505
H	-4.649	1.891	-3.761
H	-3.622	0.486	-4.169
C	-0.478	2.551	-2.175
H	-0.762	3.550	-1.783
H	0.145	2.691	-3.071
H	0.128	2.036	-1.422
C	-4.629	-0.096	-1.733
H	-4.522	-0.308	-0.665
H	-4.446	-1.022	-2.316
H	-5.656	0.243	-1.947
C	-2.268	-0.202	1.563

C	-0.746	-1.069	2.905
C	0.403	-1.581	3.517
H	1.347	-1.617	2.969
C	0.305	-2.052	4.835
H	1.193	-2.459	5.326
C	-0.913	-2.021	5.531
H	-0.960	-2.399	6.556
C	-2.079	-1.520	4.928
H	-3.029	-1.506	5.467
C	-1.977	-1.049	3.621
C	-4.301	-0.224	3.051
H	-4.689	0.446	2.272
H	-4.398	0.273	4.032
H	-4.914	-1.144	3.066
N	1.946	0.749	0.423
N	1.640	1.765	2.528
N	3.697	0.954	2.106
N	2.869	0.349	-0.537
N	0.953	-0.564	-1.637
N	2.917	-0.502	-2.759
C	2.422	1.105	1.595
C	2.479	2.264	3.607
H	1.973	2.191	4.583
H	2.740	3.330	3.436
C	3.712	1.370	3.505
H	4.649	1.891	3.761
H	3.622	0.486	4.169
C	0.478	2.551	2.175
H	0.762	3.550	1.783

H	-0.145	2.691	3.071
H	-0.128	2.036	1.422
C	4.629	-0.096	1.733
H	4.522	-0.308	0.665
H	4.446	-1.022	2.316
H	5.656	0.243	1.947
C	2.268	-0.202	-1.563
C	0.746	-1.069	-2.905
C	-0.403	-1.581	-3.517
H	-1.347	-1.617	-2.969
C	-0.305	-2.052	-4.835
H	-1.193	-2.459	-5.326
C	0.913	-2.021	-5.531
H	0.960	-2.399	-6.556
C	2.079	-1.520	-4.928
H	3.029	-1.506	-5.467
C	1.977	-1.049	-3.621
C	4.301	-0.224	-3.051
H	4.689	0.446	-2.272
H	4.398	0.273	-4.032
H	4.914	-1.144	-3.066

Table S18. Coordinates of neutral [Cu(L2)$_2$] by B3LYP/def2-SVP calculations.

Atom	x [Å]	y[Å]	z[Å]
Cu	0.000	0.000	0.000
S	0.275	-2.035	3.940
S	-0.460	0.966	-4.315
N	1.776	0.204	1.073
N	3.088	1.665	-0.228

N	4.213	0.195	1.005
N	1.768	-0.571	2.231
N	-0.428	-1.154	1.555
N	-1.642	1.158	-0.568
N	-4.042	1.525	-0.311
N	-2.710	1.701	1.461
N	-1.728	1.279	-1.952
N	0.289	0.032	-1.964
C	2.958	0.630	0.664
C	4.460	1.793	-0.692
H	4.754	2.853	-0.750
H	4.587	1.349	-1.698
C	5.240	1.022	0.383
H	6.036	0.391	-0.046
H	5.711	1.695	1.126
C	2.051	2.540	-0.715
H	1.807	2.348	-1.774
H	2.375	3.592	-0.616
H	1.137	2.395	-0.127
C	4.612	-0.956	1.790
H	5.308	-0.643	2.589
H	5.132	-1.699	1.156
H	3.727	-1.402	2.253
C	0.619	-1.147	2.423
C	-1.525	-1.824	2.048
C	-2.756	-2.005	1.392
H	-2.893	-1.591	0.391
C	-3.775	-2.724	2.025
H	-4.728	-2.867	1.509

C	-3.588	-3.274	3.301
H	-4.393	-3.838	3.779
C	-2.365	-3.111	3.966
H	-2.211	-3.544	4.958
C	-1.349	-2.390	3.341
C	-2.741	1.436	0.114
C	-4.913	1.966	0.771
H	-5.842	1.373	0.785
H	-5.196	3.030	0.643
C	-4.051	1.739	2.019
H	-4.145	2.553	2.755
H	-4.300	0.787	2.529
C	-4.612	1.276	-1.620
H	-5.128	2.181	-1.989
H	-5.354	0.458	-1.567
H	-3.811	1.023	-2.321
C	-1.545	1.864	2.294
H	-1.379	0.996	2.956
H	-1.664	2.765	2.922
H	-0.655	1.985	1.667
C	-0.699	0.759	-2.551
C	1.264	-0.359	-2.853
C	2.410	-1.117	-2.554
H	2.576	-1.452	-1.528
C	3.307	-1.444	-3.576
H	4.194	-2.039	-3.341
C	3.079	-1.033	-4.898
H	3.788	-1.300	-5.685
C	1.937	-0.285	-5.216

H	1.751	0.033	-6.245
C	1.045	0.047	-4.199

Table S19. Coordinates of the triplet state of $[Cu(L1)_2]^+$ by B3LYP/def2-SVP calculations.

Atom	x [Å]	y[Å]	z[Å]
Cu	0.000	0.000	0.000
N	-1.983	1.052	-0.349
N	-1.699	2.309	-2.319
N	-3.790	1.679	-1.853
N	-2.884	0.669	0.579
N	-1.086	-0.579	1.554
N	-3.066	-0.515	2.639
C	-2.496	1.643	-1.431
C	-2.519	3.015	-3.298
H	-2.086	2.950	-4.308
H	-2.596	4.087	-3.030
C	-3.868	2.301	-3.178
H	-4.724	2.990	-3.247
H	-3.992	1.524	-3.955
C	-0.360	2.784	-2.045
H	-0.372	3.805	-1.617
H	0.215	2.808	-2.983
H	0.145	2.110	-1.345
C	-4.892	0.840	-1.396
H	-4.568	-0.195	-1.218
H	-5.660	0.836	-2.183
H	-5.339	1.224	-0.469
C	-2.347	-0.100	1.530
C	-0.976	-1.326	2.702

C	0.106	-2.057	3.219
H	1.056	-2.089	2.681
C	-0.080	-2.734	4.421
H	0.743	-3.315	4.846
C	-1.316	-2.695	5.105
H	-1.423	-3.243	6.044
C	-2.405	-1.978	4.607
H	-3.359	-1.957	5.138
C	-2.218	-1.297	3.399
C	-4.444	-0.187	2.946
H	-4.810	0.517	2.190
H	-4.516	0.283	3.940
H	-5.074	-1.092	2.937
N	1.983	1.052	0.349
N	1.699	2.309	2.319
N	3.790	1.679	1.853
N	2.884	0.669	-0.579
N	1.086	-0.579	-1.554
N	3.066	-0.515	-2.639
C	2.496	1.643	1.431
C	2.519	3.015	3.298
H	2.086	2.950	4.308
H	2.596	4.087	3.030
C	3.868	2.301	3.178
H	4.724	2.990	3.247
H	3.992	1.524	3.956
C	0.360	2.784	2.045
H	0.372	3.805	1.617
H	-0.215	2.808	2.983

H	-0.145	2.110	1.345
C	4.892	0.840	1.396
H	4.568	-0.195	1.218
H	5.660	0.836	2.183
H	5.339	1.224	0.469
C	2.347	-0.100	-1.530
C	0.976	-1.326	-2.702
C	-0.106	-2.057	-3.219
H	-1.056	-2.089	-2.681
C	0.080	-2.734	-4.421
H	-0.743	-3.315	-4.846
C	1.316	-2.695	-5.105
H	1.423	-3.243	-6.044
C	2.405	-1.978	-4.607
H	3.359	-1.957	-5.138
C	2.218	-1.297	-3.399
C	4.444	-0.187	-2.946
H	4.810	0.517	-2.190
H	4.516	0.283	-3.940
H	5.074	-1.092	-2.937

Table S20. Coordinates of the triplet state of $[Cu(L2)_2]^+$ by B3LYP/def2-SVP calculations.

Atom	x [Å]	y[Å]	z[Å]
Cu	0.000	0.000	0.000
S	0.448	-2.166	3.877
S	-0.778	0.853	-4.311
N	1.774	0.350	1.198
N	3.029	2.055	0.151
N	4.177	0.659	1.445

N	1.775	-0.431	2.300
N	-0.313	-1.258	1.532
N	-1.615	1.347	-0.529
N	-3.918	2.087	-0.248
N	-2.520	2.208	1.475
N	-1.757	1.429	-1.869
N	0.108	-0.034	-2.002
C	2.944	0.970	0.967
C	4.424	2.422	-0.077
H	4.552	3.515	-0.040
H	4.759	2.072	-1.072
C	5.140	1.698	1.069
H	6.095	1.249	0.759
H	5.339	2.369	1.925
C	1.976	2.663	-0.630
H	2.085	2.423	-1.703
H	2.013	3.759	-0.514
H	0.997	2.304	-0.295
C	4.577	-0.392	2.369
H	4.401	-0.112	3.420
H	5.653	-0.573	2.222
H	4.030	-1.319	2.170
C	0.680	-1.172	2.428
C	-1.306	-2.114	1.944
C	-2.476	-2.428	1.225
H	-2.641	-1.974	0.245
C	-3.388	-3.320	1.779
H	-4.296	-3.575	1.228
C	-3.154	-3.908	3.038

H	-3.883	-4.610	3.450
C	-1.999	-3.614	3.765
H	-1.820	-4.077	4.738
C	-1.080	-2.717	3.214
C	-2.646	1.858	0.167
C	-4.666	2.797	0.793
H	-5.688	2.399	0.878
H	-4.738	3.875	0.552
C	-3.822	2.541	2.046
H	-3.749	3.422	2.702
H	-4.209	1.697	2.648
C	-4.512	1.895	-1.564
H	-4.297	2.735	-2.244
H	-5.601	1.812	-1.429
H	-4.138	0.978	-2.030
C	-1.361	2.047	2.324
H	-1.476	1.185	3.005
H	-1.224	2.955	2.934
H	-0.464	1.890	1.716
C	-0.831	0.756	-2.542
C	0.919	-0.598	-2.958
C	1.994	-1.476	-2.716
H	2.229	-1.764	-1.689
C	2.724	-1.961	-3.795
H	3.557	-2.645	-3.620
C	2.399	-1.590	-5.116
H	2.985	-1.987	-5.948
C	1.335	-0.726	-5.378
H	1.085	-0.446	-6.404

C	0.599	-0.234	-4.297

References

[1] Werr, M.; Kaifer, E.; Enders, M.; Asyuda, A.; Zharnikov, M.; Himmel, H.-J. A Copper(I) Complex with Two Unpaired Electrons, Synthesised by Oxidation of a Copper(II) Complex with Two Redox-Active Ligands. *Angew. Chem. Int. Ed.* **2021**, *60*, 23451–23462.

[2] Bain, G. A.; Berry, J. F. Diamagnetic Corrections and Pascal's Constants. *J. Chem. Educ.* **2008**, *85*, 532.

[3] Neese, F. Software update: The ORCA program system–Version 5.0. *WIREs Comput. Mol. Sci.* **2022**, *12*, e1606.

[4] TURBOMOLE V7.5 2020, a Development of University of Karlsruhe and Forschungszentrum Karlsruhe GmbH, 1989-2007, TURBOMOLE GmbH, since 2007; Available from `http://www.turbomole.com`.

[5] Furche, F.; Ahlrichs, R.; Hättig, C.; Klopper, W.; Sierka, M.; Weigend, F. Turbomole. *WIREs Comp. Mol. Sci.* **2014**, *4*, 91–100.

[6] Ryde, U.; Mata, R. A.; Grimme, S. Does DFT-D estimate accurate energies for the binding of ligands to metal complexes? *Dalton Trans.* **2011**, *40*, 11176–11183.

[7] Bursch, M.; Caldeweyher, E.; Hansen, A.; Neugebauer, H.; Ehlert, S.; Grimme, S. Understanding and quantifying London dispersion effects in organometallic complexes. *Acc. Chem. Res.* **2019**, *52*, 258–266.

[8] Zhao, Q.; Zhang, X.; Martirez, J. M. P.; Carter, E. A. Benchmarking an embedded adaptive sampling configuration interaction method for surface reactions: H2 desorption from and CH4 dissociation on Cu (111). *J. Chem. Theory Comput.* **2020**, *16*, 7078–7088.
